# RIM-Binding Protein 2 Promotes a Large Number of Ca_V_1.3 Ca^2+^-Channels and Contributes to Fast Synaptic Vesicle Replenishment at Hair Cell Active Zones

**DOI:** 10.3389/fncel.2017.00334

**Published:** 2017-11-02

**Authors:** Stefanie Krinner, Tanvi Butola, SangYong Jung, Carolin Wichmann, Tobias Moser

**Affiliations:** ^1^Institute for Auditory Neuroscience and InnerEarLab, University Medical Center Göttingen, Göttingen, Germany; ^2^Collaborative Research Center, University of Göttingen, Göttingen, Germany; ^3^IMPRS Molecular Biology, Göttingen Graduate School for Neuroscience and Molecular Biosciences, University of Göttingen, Göttingen, Germany; ^4^IMPRS Neuroscience, Göttingen Graduate School for Neuroscience and Molecular Biosciences, University of Göttingen, Göttingen, Germany; ^5^Synaptic Nanophysiology Group, Max Planck Institute for Biophysical Chemistry, Göttingen, Germany; ^6^DFG-Research Center for Nanoscale Microscopy and Molecular Physiology of the Brain, University of Göttingen, Göttingen, Germany; ^7^Neuromodulation and Neurocircuitry Group, Singapore Bioimaging Consortium (SBIC), Biomedical Sciences Institutes (BMSI), Agency for Science Technology and Research (A∗STAR), Singapore, Singapore; ^8^Molecular Architecture of Synapses Group, Institute for Auditory Neuroscience and InnerEarLab, University Medical Center Göttingen, Göttingen, Germany; ^9^Center for Biostructural Imaging of Neurodegeneration, University Medical Center Göttingen, Göttingen, Germany

**Keywords:** RIM-BP, calcium, exocytosis, ribbon synapse, cochlea, electron microscopy, STED microscopy

## Abstract

Ribbon synapses of inner hair cells (IHCs) mediate high rates of synchronous exocytosis to indefatigably track the stimulating sound with sub-millisecond precision. The sophisticated molecular machinery of the inner hair cell active zone realizes this impressive performance by enabling a large number of synaptic voltage-gated Ca_V_1.3 Ca^2+^-channels, their tight coupling to synaptic vesicles (SVs) and fast replenishment of fusion competent SVs. Here we studied the role of RIM-binding protein 2 (RIM-BP2)—a multidomain cytomatrix protein known to directly interact with Rab3 interacting molecules (RIMs), bassoon and Ca_V_1.3—that is present at the inner hair cell active zones. We combined confocal and stimulated emission depletion (STED) immunofluorescence microscopy, electron tomography, patch-clamp and confocal Ca^2+^-imaging, as well as auditory systems physiology to explore the morphological and functional effects of genetic RIM-BP2 disruption in constitutive RIM-BP2 knockout mice. We found that RIM-BP2 (1) positively regulates the number of synaptic Ca_V_1.3 channels and thereby facilitates synaptic vesicle release and (2) supports fast synaptic vesicle recruitment after readily releasable pool (RRP) depletion. However, Ca^2+^-influx—exocytosis coupling seemed unaltered for readily releasable SVs. Recordings of auditory brainstem responses (ABR) and of single auditory nerve fiber firing showed that RIM-BP2 disruption results in a mild deficit of synaptic sound encoding.

## Significance statement

The inner hair cell synapse provides great access for biophysical analysis of individual ribbon–type active zones. Here, we studied the role of RIM-binding protein 2 (RIM-BP2), a multi-domain protein that is part of the cytomatrix of the inner hair cell presynaptic active zone and was previously shown to directly interact with Ca_V_1.3 Ca^2+^-channels, RIMs and bassoon. We find evidence that RIM-BP2 (1) positively regulates the number of synaptic Ca^2+^-channels—the key regulators of synaptic vesicle exocytosis and (2) contributes to fast synaptic vesicle recruitment after depletion of the readily releasable pool (RRP) of vesicles. This way RIM-BP2 contributes to normal sound encoding at the inner hair cell synapse.

## Introduction

Synaptic transmission of sound information from inner hair cells (IHCs) to the auditory nerve is a fundamental step in hearing. It is this first synapse of the auditory pathway, where incoming sounds are encoded at high rates and with sub-millisecond precision (Safieddine et al., 2012; Wichmann and Moser, 2015; Moser and Vogl, 2016; Reijntjes and Pyott, 2016). Such vivid and precise signaling employs a large readily releasable pool (RRP) of synaptic vesicles (SVs) and involves a tight control of SV exocytosis by voltage-gated Ca_V_1.3 Ca^2+^-channels. In fact, the release of each RRP SV seems to be under the control of only few Ca^2+^-channels in nanometer proximity: Ca^2+^-nanodomain-like control of exocytosis (Brandt et al., 2005; Goutman and Glowatzki, 2007; Graydon et al., 2011; Kim et al., 2013; Wong et al., 2014; Pangršič et al., 2015), although Ca^2+^-microdomain like control of exocytosis has been proposed for basal IHCs in the mature gerbil cochlea (Johnson et al., 2017). A peculiar feature of inner hair cell ribbon synapses is the sustained release of hundreds of SVs/s from a single active zone (AZ) that enables continued sound-driven firing at hundreds of Hz in the postsynaptic spiral ganglion neuron (SGN). This requires efficient SV-replenishment into the RRP, which likely includes a molecular coupling of newly recruited SVs to nearby Ca^2+^-channels.

The number of Ca^2+^-channels and the fusion of each of the ~10–20 readily releasable SVs is highly regulated by the proteins forming the presynaptic cytomatrix of the active zone (CAZ). In neuronal synapses, bassoon, piccolo, liprin-α, Munc13s/CAPS, CASTs/ELKSs, Rab3 interacting molecules (RIMs) and their binding proteins (RIM-BPs) form the core of the CAZ and contribute to docking and priming of SVs (Gundelfinger and Fejtova, 2012; Südhof, 2012). So far, of the above listed CAZ proteins only bassoon, RIM2α, and RIM2β were found to be functionally relevant at IHC synapses (Khimich et al., 2005; Frank et al., 2010; Jung et al., 2015). The scaffolding protein bassoon, also anchoring the synaptic ribbon (Dieck et al., 2005; Khimich et al., 2005), is involved in tethering SVs at the AZ and their replenishment as well as the regulation of the abundance and clustering of Ca^2+^-channels, thereby creating functional release sites for SVs at IHC AZs (Frank et al., 2010; Jing et al., 2013). The multidomain scaffolding protein RIM2 plays a role in tethering SVs to the presynaptic AZ as well as in the regulation of presynaptic Ca^2+^-channel abundance and clustering (Jung et al., 2015). In contrast, so far no functional requirement was found for RIM3γ (Picher et al., 2017) that is also present at IHC AZ (Jung et al., 2015). Additionally, IHC ribbon synapses seem to operate without priming factors of the Munc-13 and CAPS family (Vogl et al., 2015) corroborating the notion of an unconventional molecular machinery at the IHC AZ (Strenzke et al., 2009; Nouvian et al., 2011).

So far the identity of putative molecular linkers between Ca_V_1.3 Ca^2+^-channels and vesicular release sites at the IHC AZ has not been elucidated: the Ca^2+^-nanodomain-like control of exocytosis was not obviously affected when disrupting bassoon (Frank et al., 2010) or RIM2α (Jung et al., 2015). RIM-BPs are candidate molecular linkers: on the one hand, they interact with RIM and therefore can connect to SVs via the RIM/Rab3 interaction and, on the other hand, bind the pore-forming Ca_V_1.3α subunit (Hibino et al., 2002; Liu et al., 2011; Acuna et al., 2015; Müller et al., 2015). Moreover, RIM-BP also binds to bassoon (Davydova et al., 2014) and, hence, may serve as an interaction node linking components of the CAZ to Ca^2+^-channels and SVs. Finally, recent work on the calyx of Held (Acuna et al., 2015) and hippocampal neurons (Grauel et al., 2016) supports such a role at central nervous system synapses. Hence, investigating the expression of RIM-BPs at ribbon synapses and the functional role they might play as part of the unconventional synaptic machinery of hair cells (Pangršič et al., 2012) is an important task. Here, we used a multidisciplinary approach combining state-of-the-art techniques to study IHC synapses of constitutive RIM-BP2 knockout mice from the molecular to the auditory systems level. High- and super-resolution microscopy of Ca_V_1.3 immunofluorescence together with functional analysis by patch-clamp and Ca^2+^-imaging report a reduction and altered spatial organization of synaptic Ca^2+^-channels in RIM-BP2-deficient IHCs. Beyond the ensuing reduction of IHC exocytosis, we find evidence for an impaired SV-replenishment in the absence of RIM-BP2. Unexpectedly, the functional coupling of Ca^2+^-influx to RRP exocytosis seemed unaltered in RIM-BP2-deficient IHCs. Moreover, using systems physiology we found only a modest impairment of sound encoding in SGNs.

## Materials and methods

### Animals

*RIM-BP2* knockout mice (*RIM-BP2*^−/−^) and their wild-type littermates *RIM-BP2* (*RIM-BP2*^+/+^) (Grauel et al., 2016) of either sex were used for experiments. For immunofluorescence microscopy, electron tomography, patch-clamp, and confocal Ca^2+^-imaging all mice were examined at the age of 2–3 weeks (i.e., after hearing-onset). Systems physiology and extracellular recordings from single SGNs were carried out at the age of 8–10 weeks. All experiments complied with national animal care guidelines and were approved by the University of Göttingen board for animal welfare and the animal welfare office of the state of Lower Saxony.

### Immunohistochemistry, confocal, and 2-color stimulated emission depletion (STED) microscopy

Freshly dissected apical turns of the organs of Corti of 3 week old *RIM-BP2*^+/+^ and *RIM-BP2*^−/−^ mice were either fixed with 4% formaldehyde (FA) in phosphate buffered saline (PBS) on ice for 10 min or with methanol at −20°C for 2 min. Thereafter, the tissue was washed 3 × 10 min in PBS and incubated for 1 h in goat serum dilution buffer (GSDB: 16% normal goat serum, 450 mM NaCl, 0.3% Triton X-100, 20 mM phosphate buffer, pH 7.4) in a wet chamber at room temperature. Primary antibodies were dissolved in GSDB and applied overnight at 4°C in a wet chamber. After washing 3 × 10 min (wash buffer: 450 mM NaCl, 20 mM phosphate buffer, 0.3% Triton X-100) the tissue was incubated with secondary antibodies in GSDB in a wet light-protected chamber for 1 h at room temperature. Then, the samples were washed 3 × 10 min in wash buffer and 1 × 10 min in 5 mM phosphate buffer, placed onto the glass microscopy slides with a drop of fluorescence mounting medium (Mowiol) and covered with thin glass coverslips. The following antibodies were used: mouse-IgG1-anti-CtBP2 (also recognizing the ribbon protein RIBEYE, BD Biosciences, 1:200), guinea pig-anti-Synapsin1/2 (Synaptic Systems, 1:500), rabbit-anti-Ca_V_1.3 (Alomone Labs, 1:50), guinea pig-anti-bassoon (Synaptic Systems, 1:500), rabbit-anti-GluA2/3 (Chemicon, 1:200), rabbit-anti-RIM-BP2 (Synaptic Systems, 1:200) as well as secondary AlexaFluor568- and AlexaFluor647-labeled goat-anti-mouse and goat-anti-rabbit antibodies (Invitrogen, 1:200) and STAR580-, and Star635P-labeled goat-anti-mouse, goat-anti-rabbit and goat-anti-guinea pig antibodies (Abberior, 1:50). Confocal images were acquired using a laser scanning confocal microscope (Leica TCS SP5, Leica Microsystems GmbH, Mannheim, Germany) equipped with 561 and 633 nm lasers for excitation and a 63x oil immersion objective (1.4 NA, Leica). STED images were acquired using a 2-color STED microscope (Abberior Instruments, Göttingen, Germany) equipped with 561 and 640 nm excitation lasers, a 775 nm laser for STED (1.2 W) and a 100x oil immersion objective (1.4 NA, Olympus). Samples were treated in parallel and images were acquired in parallel, using same laser power, gain, and microscope settings. Images were analyzed using ImageJ, Igor Pro, OriginPro and Imaris software, and assembled for display in Adobe Illustrator software.

### Electron tomography

Embedding of apical turns of the organ of Corti of 3-week-old *RIM-BP*^+/+^ and *RIM-BP*^−/−^ mice was performed as described previously (Wong et al., 2014). Following conventional embedding and trimming of the blocks, 250 nm sections were obtained for electron tomography with an Ultracut E microtome (Reichert-Jung). Sections were poststained for 40 min using uranyl acetate-replacement solution and subsequently 30 s with lead citrate. Electron tomography, tomogram generation and rendering were essentially performed as described previously (Strenzke et al., 2016) and references therein. Tomograms were generated using the IMOD software program etomo. Rendering of 3-dimensional (3D) models and quantitative image analysis was performed blinded using 3dmod. The size of synaptic ribbons and presynaptic densities (PD) was determined from the surface area of the reconstructed 3D objects. For “ribbon-associated” vesicles (RA-SVs) the first row of vesicles around the synaptic ribbon within a distance of 80 nm was counted. For “membrane-proximal” vesicles (MP-SVs) the vesicles with a maximum lateral distance (membrane to membrane) of 100 nm from the PD and ≤ 50 nm from the presynaptic plasma membrane were counted (Jung et al., 2015).

### Patch-clamp and confocal Ca^2+^-imaging of inner hair cells

IHCs from apical coils of freshly dissected organs of Corti of 2-week-old *RIM-BP2*^+/+^ and *RIM-BP2*^−/−^ mice were patch-clamped at room temperature (22–24°C) as described (Moser and Beutner, 2000). For perforated-patch-clamp recordings of whole-cell Ca^2+^-current and exocytosis the pipette solution contained (in mM): 130 Cs-gluconate, 10 TEA-Cl, 10 4-AP, 10 HEPES, 1 MgCl_2_, amphotericin B (300 μg/ml), pH 7.2. For confocal Ca^2+^-imaging we performed ruptured-patch-clamp recordings with the solution described above excluding amphotericin but including 4 mM Mg-ATP, 0.3 mM Na-GTP, 1 mM EGTA, 0.4 mM Fluo-4FF (Invitrogen), and 0.01 mM carboxytetramethylrhodamine(TAMRA)-conjugated dimeric RIBEYE-binding peptide (Francis et al., 2011). The extracellular solution contained (in mM): 110 NaCl, 35 TEA-Cl, 2.8 KCl, 2 CaCl_2_, 1 MgCl_2_, 10 NaOH-HEPES, 11.3 D-glucose, pH 7.3. EPC-10 amplifiers (HEKA Elektronik, Lambrecht, Germany) controlled by “Patchmaster” software (HEKA) were used for measurements. All voltages were corrected for liquid junction potentials (14 mV). Currents were leak-corrected using a p/10 protocol. Current-voltage relationships (IVs) were calculated from the last 8 ms of currents evoked by step depolarizations to various potentials. From these, fractional activation curves were calculated by first calculating the conductance of the calcium current as $G\left(V\right)=\frac{I_{Ca}}{\left(V-V_{rev}\right)}$, where *V* is the command potential and *V*_rev_ is the reversal potential of the current obtained by extrapolating a line fit from 6 to 26 mV. After normalizing these traces to the maximum conductance in the range of −20 to +10 mV, they were fit with a Boltzmann equation $G_{n}\left(V\right)=\frac{G_{n,\;max}}{1+e^{\frac{V_{half}-V}{k}}}$ with G_n,max_, as the maximum conductance, *V* as the command potential, *V*_*half*_ as the voltage of half-maximal activation, and k as the slope factor. I_Ca_ inactivation kinetics were calculated from fitting a single exponential function to the last 195 ms of I_Ca_ traces during 200 ms depolarizations to −14 mV (corrected for liquid junction potential). For membrane capacitance (*C*_*m*_) measurements, IHCs were stimulated by depolarizations to −14 mV with intervals of 60–120 s to allow for recovery of IHC exocytosis. Two pulse protocols were used: (i) single depolarizations of different durations and (ii) paired depolarizations with variable inter-pulse interval. We measured *C*_*m*_ changes (Δ*C*_*m*_) using the Lindau-Neher technique (Lindau and Neher, 1988) as previously described (Moser and Beutner, 2000). Briefly, the exocytic Δ*C*_*m*_ was quantified as the difference of the averaged *C*_*m*_ 400 ms before and after the depolarization. To avoid impact of *C*_*m*_-transients related to conductance or gating of ion channels on Δ*C*_*m*_ estimation (Moser and Beutner, 2000; Neef et al., 2007) we skipped the first 100 ms (single depolarizations) or 25 ms (paired depolarizations) of post-depolarization *C*_*m*_ for estimating the average. Mean Δ*C*_*m*_ and Ca^2+^-current estimates present grand averages calculated from the mean estimates of individual IHCs, where each depolarization was repeated 2–3 times. This avoided dominance of IHCs contributing more sweeps.

The apparent Ca^2+^-cooperativity *m* of exocytosis was addressed via gradual reduction of the number of open Ca^2+^-channels by slow wash-in (0.5 ml/min) of the dihydropyridine L-type Ca^2+^-channel antagonist isradipine (10 μM) in the extracellular solution (here using 5 mM CaCl_2_ and 104 mM NaCl). The exocytic Δ*C*_*m*_ and corresponding I_Ca_ evoked by 20 ms depolarizations (to −14 mV, corrected for liquid junction potential) were recorded while slowly reducing the number of unblocked Ca^2+^-channels and Δ*C*_*m*_ to the Ca^2+^-current integral (Q_Ca_) for each individual IHC and fit by a power function Δ*C*_*m*_ = *A (Q*_*Ca*_*)*^*m*^, where the power *m* describes the apparent Ca^2+^-cooperativity of synaptic vesicle exocytosis.

For confocal Ca^2+^-imaging we used the low affinity Ca^2+^-indicator Fluo-4FF (K_D_ = 10 μM, Invitrogen) and also loaded the IHCs with TAMRA-labeled RIBEYE-binding peptide for visualization of the ribbons (Zenisek, 2008; Frank et al., 2009). Line scans (0.7 kHz) across the center of fluorescently labeled AZs were performed during IHC depolarization (20 ms to −7 mV, three repeats per AZ) to measure Fluo-4FF fluorescence changes as a consequence of synaptic Ca^2+^-influx (Frank et al., 2009). For quantification of line-scans, the Fluo-4FF ΔF-signal was calculated as the average evoked change in intensity from three peak-centered pixels that showed the maximal fluorescence intensity (F_max_) over the last 10 ms of depolarization. ΔF was normalized to F_0_, for which we used the fluorescence intensity of the same three peak-centered pixels before IHC depolarization.

### Systems physiology: auditory brainstem responses (ABR), distortion product otoacoustic emissions (DPOAE) and extracellular recordings from spiral ganglion neurons

ABR, DPOAE, and extracellular recordings from single SGNs were performed as described before (Jing et al., 2013; Strenzke et al., 2016) on 8–10 week-old mice. For extracellular recordings from individual SGNs, mice were anesthetized by i.p. injection of urethane (1.32 mg/kg), xylazine (5 mg/kg), and buprenorphine (0.1 mg/kg), a tracheostomy was performed, and the mice were then placed in a stereotactic system. After partial removal of the occipital bone and cerebellum to expose the anteroventral cochlear nucleus (AVCN), a glass microelectrode was advanced through the posterior AVCN portion to reach the auditory nerve where it enters the AVCN. Acoustic stimulation was provided by an open field Avisoft ScanSpeak Ultrasonic Speaker (Avisoft Bioacoustics), and “putative” auditory nerve fibers (formed by the central SGN axons) were identified and distinguished from cochlear nucleus neurons based on their stereotactic position (>1.1 mm from the surface of the cochlear nucleus), spontaneous and noise-burst induced firing, peristimulus time histogram (PSTH), regularity of firing, and first spike latency. Recordings were performed using TDT system III hardware and an ELC-03XS amplifier (NPI electronics).

Data analysis and statistical tests were done in Igor Pro for electrophysiology, Ca^2+^-imaging, and immunohistochemistry. Normality of distribution was tested with Jarque-Bera test and variances were compared with *F*-test. Unpaired, two-tailed Wilcoxon rank test (Mann-Whitney test) was used to compare non-normal or data with unequal variances. Else, unpaired, two-tailed Student's *t*-test were used. For comparing the variance of the ΔF/F_0_ estimates for synaptic Ca^2+^-signals we used the Brown-Forsythe-modified Levene test. The distance of MP-SVs from the plasma membrane was compared between genotypes in the ranges of 0–25 and 26–50 nm by the Kolmogorov–Smirnov test. Data analysis for systems physiology was done using waveform-based spike detection using custom-written MATLAB software. Further statistical analysis was done in Igor Pro as described above for all parameters except spontaneous rate distributions, which were compared using Mann Whitney *U*-test in GraphPad Prism.

## Results

### RIM-BP2 forms stripe-shaped clusters at inner hair cell active zones

**Figure 1:**
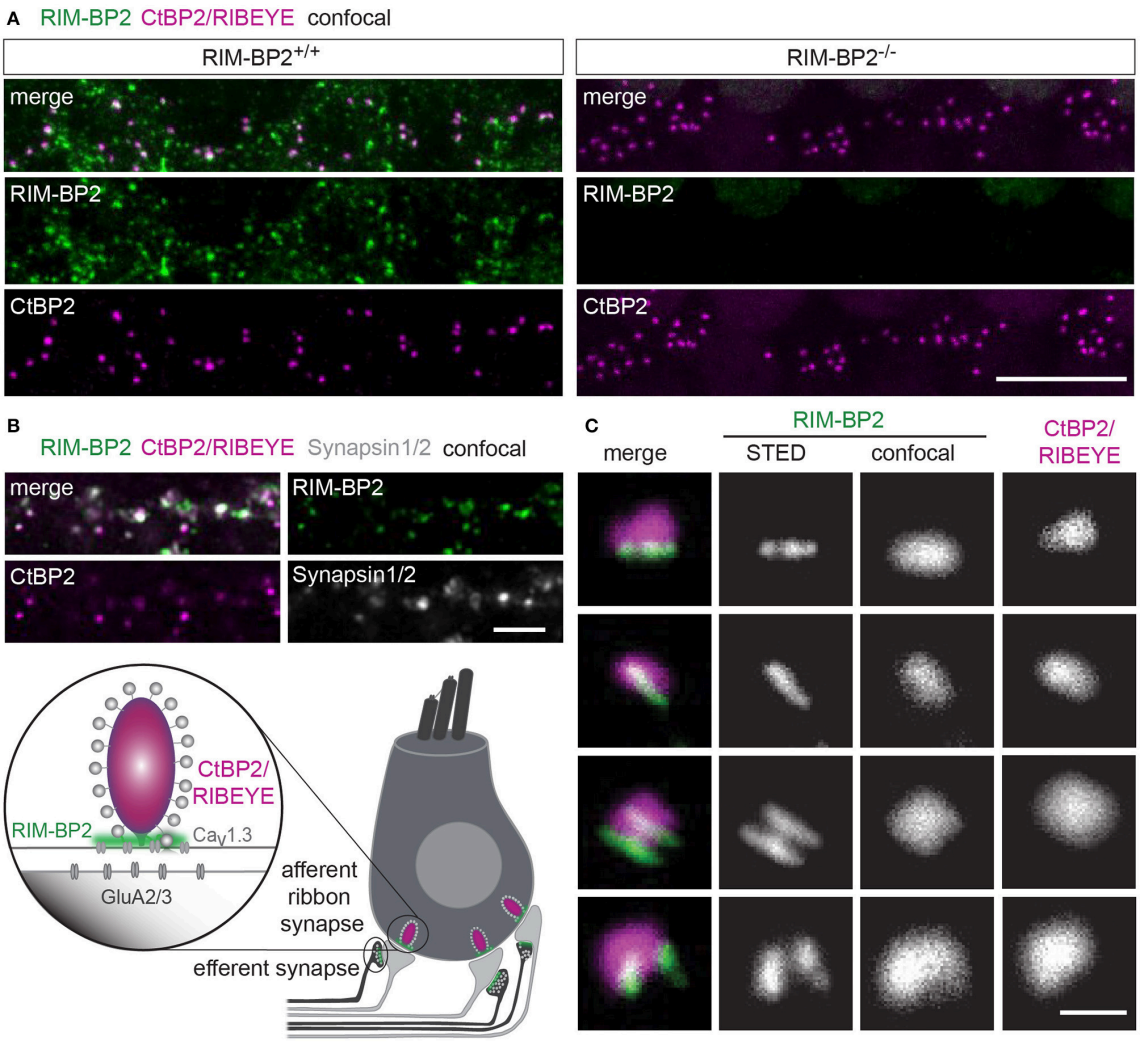
RIM-BP2 forms stripe like clusters at the base of the synaptic IHC ribbon. **(A)** Expression analysis of RIM-BP2 in IHCs: Maximum projections of confocal stacks after immunohistochemistry of whole-mount explants of apical organs of Corti. RIM-BP2 immunofluorescence (green) co-localizes with presynaptic ribbons (CtBP2/RIBEYE, magenta) in *RIM-BP2*^+/+^ IHCs and is absent in parallel processed littermate *RIM-BP2*^−/−^ IHCs, indicating specificity of the immunolabeling. Additional RIM-BP2 fluorescence in *RIM-BP2*^+/+^ IHCs most likely localizes to presynaptic efferent terminals **(B)**. Scale bar: 10 μm. **(B)** Top: Maximum projections of confocal stacks after triple-immunostaining of whole-mount explants of apical organs of Corti. RIM-BP2 immunofluorescence (green) co-localizes with either presynaptic ribbons (CtBP2/RIBEYE, magenta) or Synapsin1/2 (gray) marked presynaptic terminals of efferent lateral olivocochlear neurons. Scale bar 5 μm. Bottom: Illustration of a mature murine IHC: Afferent type I SGNs innervate ribbon synapses at the IHC base. Efferent synapses of the lateral olivocochlear neurons form projections onto type I SGN synapses nearby the IHC. Location of presynaptic ribbons (CtBP2/RIBEYE) is highlighted in magenta. RIM-BP2 (green) forms clusters at the presynaptic density at the base of the synaptic ribbon and at the AZs of efferent presynaptic terminals. **(C)** Nanoscale organization of RIM-BP2: 2-color STED microscopy of individual active zones from whole mount explants of apical organs of Corti after immunohistochemistry resolved a mostly stripe or double-stripe-like expression pattern of RIM-BP2 (green) at the ribbon (CtBP2/RIBEYE, magenta). Simultaneously acquired confocal images could not resolve single or double stripes of RIM-BP2. Single XY-sections, scale bar: 500 nm. **(A–C)** Age of mice: p21.

First, we studied the localization of RIM-BP2 at IHC AZs using immunolabeling and confocal microscopy of mouse IHCs. RIM-BP2 immunofluorescence at CtBP2/RIBEYE-marked ribbon-occupied AZs of *RIM-BP2*^+/+^ IHCs and was absent from the basal pole of IHCs of *RIM-BP2*^−/−^ IHCs confirmed specificity of the immunolabeling (Figure 1A). In addition, some RIM-BP2 immunofluorescence spots were found near IHCs but away from the ribbons, most likely reflecting their presence in conventional presynaptic terminals formed by efferent lateral olivocochlear neurons onto the postsynaptic boutons of the afferent SGNs near the base of the IHCs (connectivity illustrated in Figure 1B). This notion was confirmed by triple antibody staining for RIM-BP2, CtBP2/RIBEYE and Synapsin1/2, marking the presynaptic terminals of efferent lateral olivocochlear neurons (Safieddine and Wenthold, 1999) (Figure 1B). RIM-BP2 immunofluorescence co-localized with either CtBP2/RIBEYE or Synapsin1/2, but was not visible outside the marked presynaptic regions, suggesting specific localization of RIM-BP2 to AZs of IHCs and presynaptic terminals of efferent lateral olivocochlear neurons. Using 2D 2-color STED microscopy we observed that RIM-BP2 immunofluorescence at the ribbon-type AZs was mostly arranged in single or double stripe-like shapes (Figure 1C). This pattern was similar for other CAZ proteins of the IHC AZ: bassoon and RIM2 both predominantly form stripe-like clusters at the base of the synaptic ribbon (Frank et al., 2010; Jung et al., 2015). Likewise, Ca_V_1.3 Ca^2+^-channel clusters at IHC AZs primarily assume single or double-stripe like shapes (Frank et al., 2010; Wong et al., 2014). Thus, the spatial organization of RIM-BP2 seems to be very similar to that of the other CAZ proteins at the IHC AZ, where these proteins form the presynaptic density and co-align with the presynaptic Ca_V_1.3 Ca^2+^-channels cluster. Unfortunately, co-immunostaining of RIM-BPs together with Ca_V_1.3 Ca^2+^-channels was not possible, since the antibodies available to us were from the same species (see section Materials and Methods).

### RIM-BP2 disruption does not affect inner hair cell afferent connectivity

**Figure 2:**
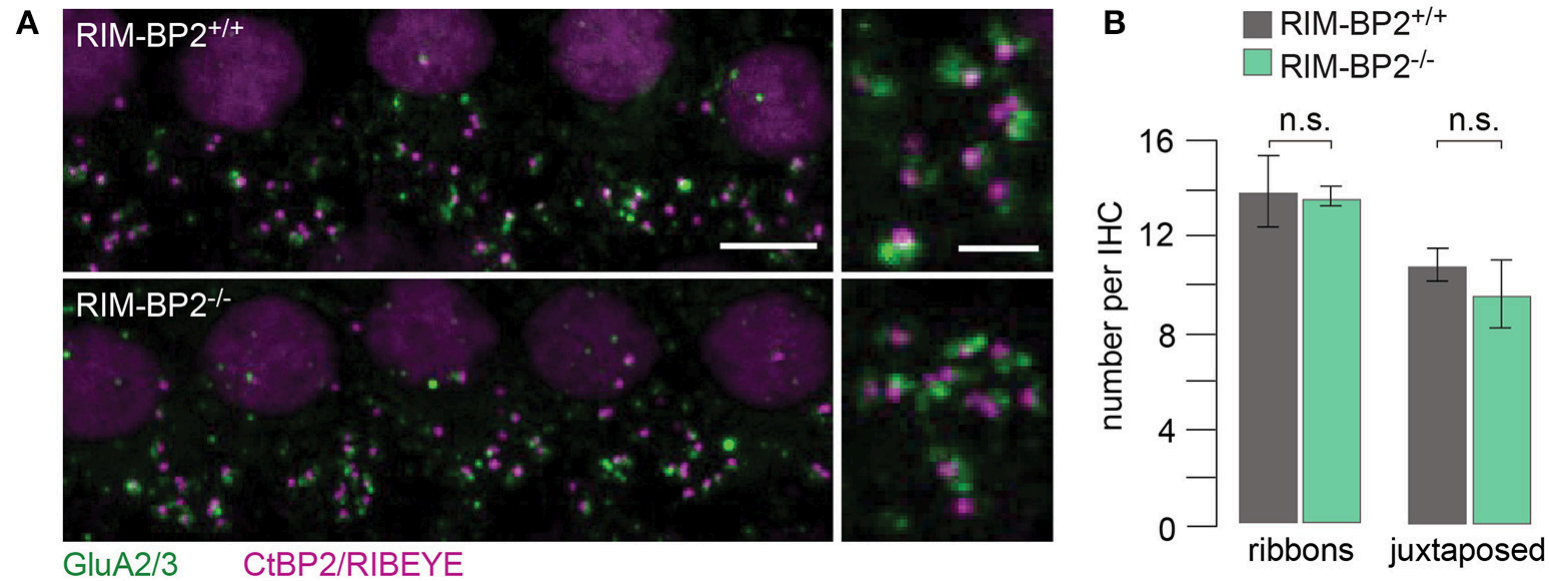
RIM-BP2 disruption does not affect afferent IHC connectivity. **(A)** Left: Maximum projections of confocal stacks from whole-mount explants from apical organs of Corti of p21 mice, immunolabeled for CtBP2/RIBEYE (magenta) and GluA2/3 (green). Scale bar: 5 μm. Right: Higher magnification shows details of synapses. Scale bar: 2 μm; age of mice: p21. **(B)** 3D analysis of fluorescently labeled IHC ribbon synapses shows that the number of ribbons (CtBP2/RIBEYE spots, *p* = 0.9) and the number of ribbon-occupied synapses (number of juxtaposed RIBEYE/CtBP2 and GluA2/3 spots, *p* = 0.6) per IHC were not altered in RIM-BP2-deficient (*RIM-BP2*^−/−^, *n* = 38, *N* = 2) IHCs compared to control (*RIM-BP2*^+/+^, *n* = 56, *N* = 2) IHCs. Data represent grand averages, mean ± SEM; Wilcoxon rank test: significance level: n.s. *p* ≥ 0.05, *n* = number of IHCs, *N* = number of mice.

Using constitutive *RIM-BP2* knockout mice (*RIM-BP2*^−/−^), we first addressed the afferent connectivity of IHCs using confocal microscopy and 3D analysis of immunolabeled synapses (Khimich et al., 2005). RIBEYE/CtBP2 (synaptic ribbon) served as a presynaptic synapse marker and GluA2/3 (postsynaptic AMPA receptors) as a postsynaptic synapse marker (Figure 2A). We found the number of ribbons (RIBEYE/CtBP2 spots) (*p* = 0.9) and ribbon-occupied synapses (number of juxtaposed RIBEYE/CtBP2 and GluA2/3 spots) (*p* = 0.6) to be comparable in *RIM-BP2*^+/+^ and *RIM-BP2*^−/−^ IHCs (Figure 2B), indicating that the afferent hair cell connectivity was unaltered.

### Disruption of RIM-BP2 reduces presynaptic Ca^2+^-influx

**Figure 3:**
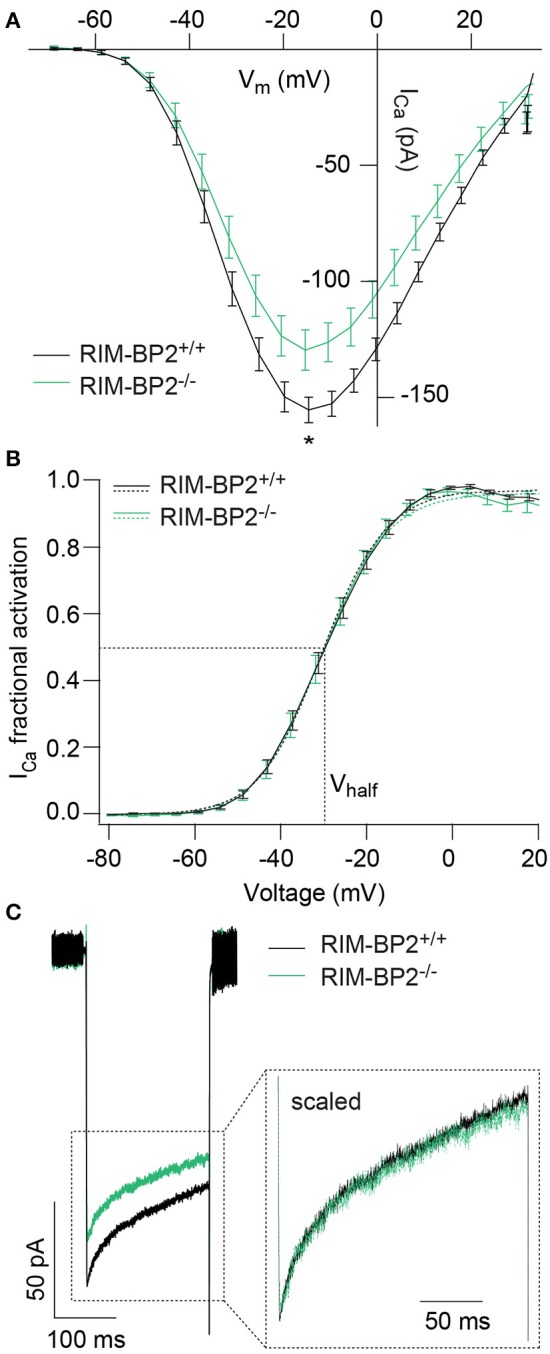
RIM-BP2 promotes voltage-dependent Ca^2+^-influx in IHCs. **(A)** Ca^2+^ current-voltage relationship of RIM-BP2-deficient (*RIM-BP2*^−/−^, *n* = 16, green) and control (*RIM-BP2*^+/+^, *n* = 17, black) IHCs. Current-voltage relationships (IVs) were calculated from the last 8 ms of currents evoked by step depolarizations to various potentials. Ca^2+^-current amplitude was significantly reduced in RIM-BP2-deficient IHCs. **(B)** Fractional activation curves of the whole-cell Ca^2+^-current: A Boltzmann function was fit to the normalized conductance curve **(B)** calculated from the Ca^2+^-current-voltage relationship **(A)**. Average fit data (dashed traces) are displayed for both genotypes (*RIM-BP2*^−/−^*, n* = 16, green and *RIM-BP2*^+/+^, *n* = 17, black). Dashed lines indicate V_half_ and the slope k, reporting the voltage of half-maximal activation of the whole-cell Ca^2+^-current and the voltage-sensitivity of Ca^2+^-influx. **(C)** Ca^2+^-current, I_Ca_ during 200-ms step depolarization to −14 mV of RIM-BP2-deficient (*RIM-BP2*^−/−^, *n* = 11, green) and control (*RIM-BP2*^+/+^, *n* = 15, black) IHCs. Direct comparison of the Ca^2+^-currents through scaling of the Ca^2+^-currents showed no difference in the inactivation of the Ca^2+^-influx. **(A–C)** Mean ± SEM and statistical *p*-values are displayed in Table 1. Data information: Data represent IHC grand averages, mean ± SEM; Significance level: ^*^p < 0.05. *n* = number of IHCs; age of mice: p14-p16.

Since RIM-BPs are known to interact with the pore-forming Ca_V_1.3α subunit (Coppola et al., 2001; Hibino et al., 2002), we next studied the effect of RIM-BP2 disruption on the presynaptic Ca^2+^-influx using perforated patch-clamp recordings of voltage-gated Ca^2+^-influx in *RIM-BP2*^−/−^ IHCs. We observed a significant reduction of the maximal whole-cell I_Ca_ amplitude in *RIM-BP2*^−/−^ IHCs (~20%) (Figure 3A, Table 1). We then analyzed the voltage-dependence of I_Ca_ activation (voltage of half-maximal activation, V_half_ and slope factor k; Figure 3B) as well as the kinetics and extent of I_Ca_ inactivation (Figure 3C), which were unaltered in *RIM-BP2*^−/−^ IHCs (Table 1). While our data seem not to support a regulation of Ca_V_1.3 gating by RIM-BP2 in IHCs, we cannot exclude a reduced open probability as cause of the diminished Ca^2+^-influx. However, we favor the simple interpretation of the reduction of Ca^2+^-influx in RIM-BP2-deficient IHCs that RIM-BP2 positively regulates the number of Ca^2+^-channels in IHCs.

**Table 1:** Summary of Ca^2+^-current (I_Ca_) data from perforated patch-clamp recordings.

	**Amplitude (pA)**	**V_half_ (mV)**	**Slope factor k**	**τ_inactivation_ (ms)**
*RIM-BP2^+/+^*	−157 ± 6 (*n* = 17)	−29.9 ± 0.9 (*n* = 17)	6.9 ± 0.1 (n = 17)	90 ± 11 (*n* = 15)
*RIM-BP2^−/−^*	−126 ± 9 (*n* = 16)	−30. ± 1.5 (*n* = 16)	6.7 ± 0.2 (*n* = 16)	98 ± 13 (*n* = 11)
*p*-value	0.02^*^	0.9	0.2	0.3
	Wilcoxon rank test	Student's *t*-test	Wilcoxon rank test	Wilcoxon rank test

*Summary of IHC grand average data from perforated patch-clamp recordings of Ca^2+^-current (I_Ca_) from RIM-BP2^+/+^ and RIM-BP2^−/−^ IHCs (Figures 3A–C). Whole-cell I_Ca_ was analyzed regarding amplitude, voltage-dependence (V_half_), Ca^2+^-channel gating (slope factor k representing voltage-sensitivity of Ca^2+^-influx) and kinetics of I_Ca_ inactivation (τ_inactivation_). Data represent IHC grand averages, mean ± SEM; n = number of IHCs; p-values and statistical test are depicted for each dataset, significance level*:

* *p < 0.05*.

### Disruption of RIM-BP2 reduces synaptic Ca_V_1.3 abundance and synaptic Ca^2+^-influx

**Figure 4:**
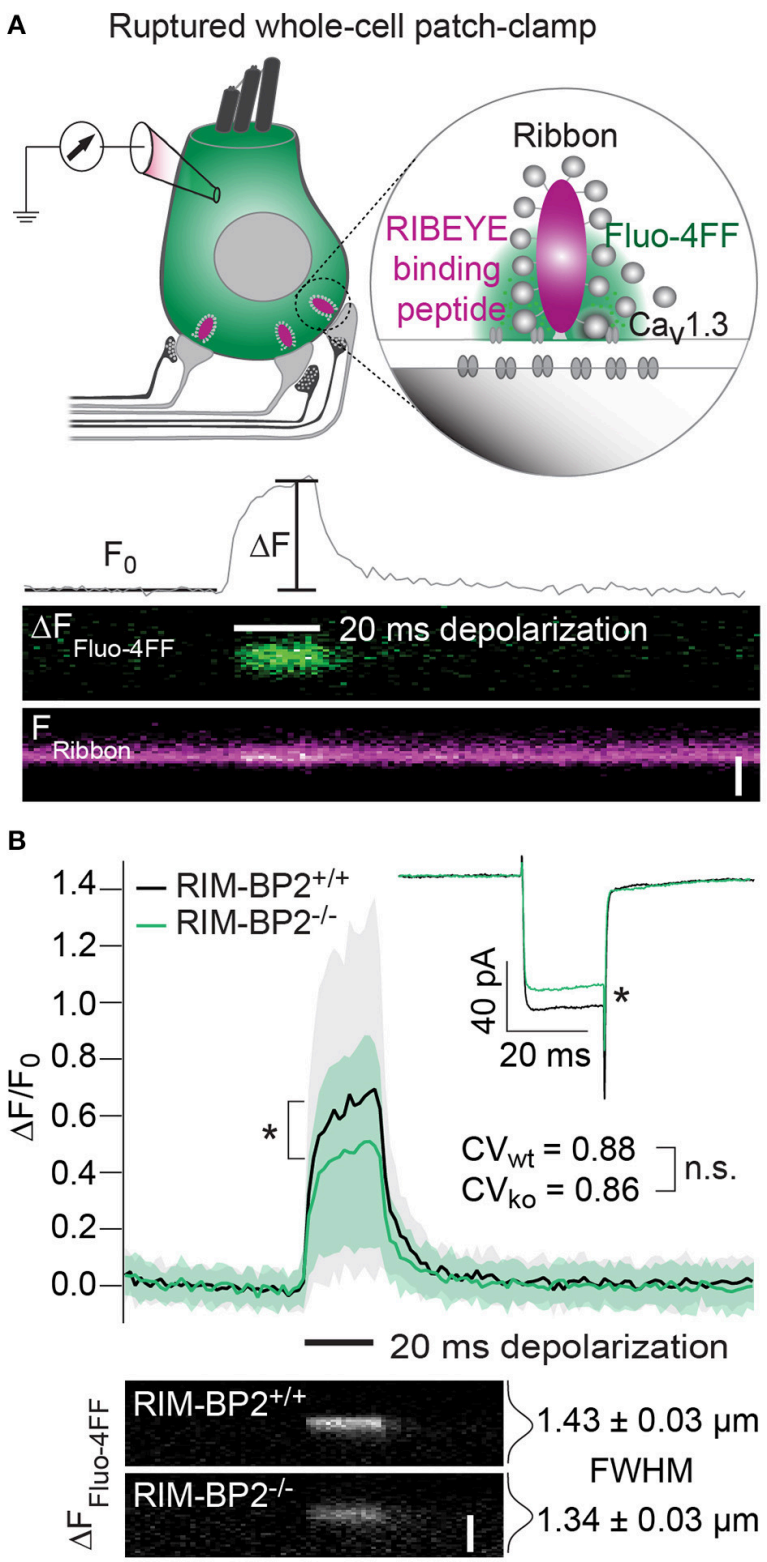
RIM-BP2 promotes the abundance of synaptic Ca^2+^-channels in IHCs. **(A)** Top: Illustration of the confocal Ca^2+^-imaging experimental design. IHCs were loaded with a fluorescently conjugated dimeric RIBEYE-binding peptide (10 μM) to visualize synaptic ribbons at the AZ, and a low affinity Ca^2+^-indicator Fluo-4FF (400 μM) to monitor synaptic Ca^2+^-influx. Bottom: Example line scan of a fluorescently labeled ribbon (F_ribbon_) and Fluo-4FF fluorescence change (ΔF_Fluo−4FF_) at an individual IHC AZ during 20 ms depolarization (black bar) and temporal profile of Fluo-4FF fluorescence at the center of the ribbon. ΔF_Fluo−4FF_ from line-scans was normalized to their baseline fluorescence F_0_ hence ΔF/F_0_. Scale bar: 2 μm. **(B)** Top: Average temporal profile of synaptic Ca^2+^-signals (ΔF/F_0_) for *RIM-BP2*^+/+^ (*n* = 41, *N* = 14, black line) and *RIM-BP2*^−/−^ (*n* = 42, *N* = 13, green line) AZs during IHC depolarizations (black bar). ΔF/F_0_ and its corresponding I_Ca_ were significantly reduced in *RIM-BP2*^−/−^ IHCs (Wilcoxon rank test, both *p* = 0.01). ΔF/F_0_ data represent IHC grand averages, mean ± *SD* (shaded area). The coefficient of variation (CV) represents synapse heterogeneity and was comparable within genotypes (modified Levene's test). Bottom: Representative Ca^2+^-signals (same intensity-scale) of *RIM-BP2*^+/+^ and *RIM-BP2*^−/−^ AZs showing comparable spatial spread of synaptic Ca^2+^ influx estimated by a Gaussian fit function (Student's *t*-test. *p* = 0.5). Scale bar: 2 μm. Data information: Data represent grand averages, mean ± SEM, unless ΔF/F_0_ (see above); Significance level: n.s. *p* ≥ 0.05, ^*^p < 0.05. *n* = number of AZs, *N* = number of IHCs; age of mice: p14-16.

Since the whole-cell recordings sum I_Ca_ of all AZs and the extrasynaptic membrane, we sought to further analyze Ca^2+^-influx at the single AZ level using laser scanning confocal Ca^2+^-imaging (Frank et al., 2009; Wong et al., 2014). Using ruptured patch-clamp, we loaded IHCs with a fluorescently labeled RIBEYE-binding peptide to visualize the location of synaptic ribbons (Zenisek et al., 2004; Ohn et al., 2016) and a low affinity Ca^2+^-indicator Fluo-4FF. Under the chosen conditions ([Ca^2+^]_e_: 2 mM, Ca^2+^ buffering: 1 mM EGTA, K_D_ of Fluo-4FF for Ca^2+^: 10 μM) we expect the Fluo-4FF fluorescence changes (ΔF/F_0_) to be a good proxy for the synaptic Ca^2+^-influx (Frank et al., 2009). Line scans across the center of fluorescently labeled AZs were performed during IHC depolarization (20 ms to −7 mV) to measure Fluo-4FF ΔF/F_0_ as a consequence of synaptic Ca^2+^-influx (Figure 4A). We found a significant reduction (~41%, *p* = 0.01) of the Fluo-4FF maximal fluorescence change (ΔF_max_/F_0_) at AZs of *RIM-BP2*^−/−^ IHCs, which exceeded the reduction of their whole-cell Ca^2+^-current (~20%, *p* = 0.01) (Figure 4B). This suggests that RIM-BP2 disruption caused a preferential loss of synaptic Ca^2+^-channels. As previously described for wild-type IHCs, the maximal ΔF/F_0_ estimates varied greatly among AZs within IHCs. This variance is likely explained by the heterogeneity of synapses regarding their AZ size and Ca^2+^-channel number (Frank et al., 2009; Wong et al., 2013; Ohn et al., 2016). We assessed the synapse heterogeneity by calculating the coefficient of variation (CV) of the ΔF_max_/F_0_ signals, which was comparable between *RIM-BP2*^−/−^ (CV = 0.86) and *RIM-BP2*^+/+^ AZs (CV = 0.88). Hence, despite the reduced synaptic Ca^2+^-influx, presynaptic heterogeneity was unchanged at *RIM-BP2*^−/−^ AZs (Figure 4B). Furthermore, we estimated the spatial extent of the synaptic Ca^2+^ signals by fitting a 1D Gaussian function to the ΔF amplitudes of the line scans, 15 ms after depolarization onset. The full width at half maximum (FWHM) at *RIM-BP2*^−/−^ AZs were not significantly different to that of *RIM-BP2*^+/+^ AZs (*RIM-BP2*^+/+^ FWHM = 1.43 ± 0.03 μm, *RIM-BP2*^−/−^ FWHM = 1.34 ± 0.03 μm, *p* = 0.5, Figure 4B). As the confocal Ca^2+^-imaging does not provide insight into the nanoscale organization of Ca^2+^-channels at the AZ we turned to STED super-resolution microscopy of Ca_V_1.3 immunofluorescence.

**Figure 5:**
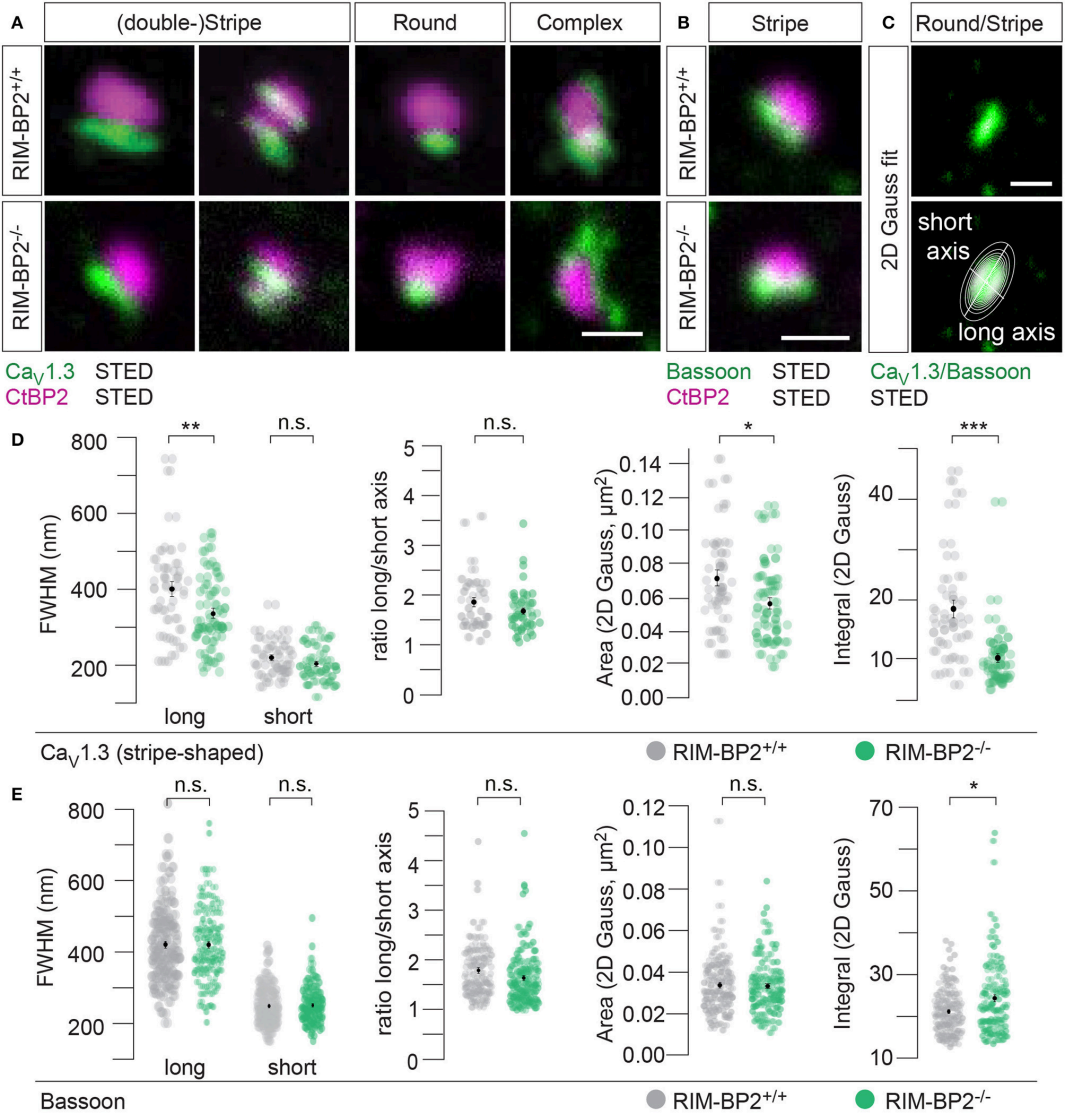
RIM-BP2 promotes synaptic Ca^2+^-channel abundance at IHC ribbon synapses downstream of bassoon. **(A,B)** XY-sections of individual AZs acquired with high-resolution 2D 2-color STED microscopy. Whole-mount explants of apical *RIM-BP2*^+/+^ and *RIM-BP2*^−/−^ organs of Corti were immunolabeled for CtBP2/RIBEYE (magenta) and Ca_V_1.3 (green, **A**) or bassoon (green, **B**). Scale bars: 500 nm. **(A)** Ca_V_1.3 clusters were subjectively classified in “(double-) stripe-like,” “round” and “complex” apparent shapes. **(C)** 2D Gaussian functions were fit to each individual STED image of “stripe-shaped” Ca_V_1.3 and bassoon clusters to approximate the cluster dimensions. Fit amplitudes and FWHM of the long and short axis (illustrated) of the Gaussian function were used for quantitative analysis in **(D,E)**. Scale bar: 500 nm. **(D,E)** Quantitative analysis of Ca_V_1.3 **(D)** and bassoon **(E)** cluster STED images described in **(C)**. Data of FWHM long and short axis, the ratio of long over short axis, cluster area and integral are displayed as individual data points (*RIM-BP2*^+/+^ in gray, *RIM-BP2*^−/−^ in green) and grand averages as mean ± SEM (black). **(D)** *RIM-BP2*^+/+^ *n* = 37, N = 2; *RIM-BP2*^−/−^ *n* = 49, *N* = 2. **(E)** *RIM-BP2*^+/+^ *n* = 127, *N* = 2; *RIM-BP2*^−/−^ *n* = 124, *N* = 2; **(D,E)** Mean ± SEM and statistical *p*-values are displayed in Table 2. Significance levels: n.s. *p* ≥ 0.05, ^*^p < 0.05, ^**^p < 0.01, ^***^p < 0.001; *n* = number of AZs, *N* = number of mice; age of mice: p21-p23.

Ca_V_1.3 Ca^2+^-channels remained clustered at ribbon-occupied AZs in *RIM-BP2*^−/−^ IHCs (Figure 5A). We subjectively classified the Ca^2+^-channel clusters according to their appearance, whereby the majority of them (66%) assumed a “stripe-like” or “double stripe-like” arrangement of Ca^2+^-channels. Only 12% of Ca^2+^-channel clusters formed “round” and 22% “complex” cluster shapes. We note that we focused on synapses seemingly lying en face in the imaging plane, and refer to apparent shapes as we did not assess the clusters with 3D STED resolution. We found the classes of apparent shapes and their representation to be very similar in *RIM-BP2*^−/−^ IHCs with a slight trend toward less stripe-shaped (62%) and more round (16%) appearance. We approximated the dimensions of “stripe-shaped” Ca^2+^-channel clusters by fitting a 2-dimensional (2D) Gaussian function to STED images of Ca_V_1.3 immunofluorescence (Figures 5A,C, quantifications Figure 5D, Table 2). The FWHM of the long axis was significantly shorter (*p* = 0.009) and the cluster area (22% reduction, *p* = 0.01) as well as the integral of the 2D Gaussian fit function (~49% of WT, *p* < 0.0001) were significantly smaller in *RIM-BP2*^−/−^ AZs (Figure 5D), whereas the short axis was unaltered (*p* = 0.1). Consequently, there was a trend toward a smaller FWHM ratio of long over short axis, which, however, did not reach statistical significance (*p* = 0.1). We interpret the calculated area and integral of the 2D Gaussian fit as a measure of Ca^2+^-channel cluster size and the integrated Ca_V_1.3 immunofluorescence as a proxy for the number of Ca^2+^-channels. However, we note that Ca_V_1.3 immunofluorescence might not relate linearly to the number of Ca^2+^-channels. In summary, our morphological STED imaging indicates a “rounder” shape, a smaller size and a halved integrated Ca_V_1.3 immunofluorescence of the Ca^2+^-channel clusters of RIM-BP2-deficient AZs, which is consistent with the 41% reduction of the ΔF_max_/F_0_ found by Ca^2+^-imaging. The same analysis was performed for bassoon clusters (Figure 5B, quantifications Figure 5E, Table 2). Here, we found no difference in the FWHM of the long axis, short axis, long/short axis ratio and cluster area between AZs of the two genotypes. However, a significant increase in bassoon cluster integral (21%) was found in *RIM-BP2*^−/−^ AZs potentially indicating a compensatory upregulation of bassoon.

**Table 2:** Summary of Ca_V_1.3 and bassoon STED image quantification data.

	**FWHM long axis (nm)**	**FWHM short axis (nm)**	**Ratio long/short axis**	**Area (μm^2^)**	**Integral (μm^2^ x a.u.)**
Ca_V_1.3 *RIM-BP2^+/+^ (n = 37, N = 2)*	403 ± 20	221 ± 8	1.85 ± 0.09	0.072 ± 0.005	18.3 ± 1.7
Ca_V_1.3 *RIM-BP2^−/−^(n = 49, N = 2)*	338 ± 13	205 ± 6	1.67 ± 0.06	0.056 ± 0.003	8.98 ± 0.9
Ca_V_1.3 *p*-value	0.009^**^	0.1	0.1	0.01^*^	7.2e-8^***^
	Wilcoxon rank test	Studen't *t*-test	Wilcoxon rank test	Studen't *t*-test	Wilcoxon rank test
Bassoon *RIM-BP2^+/+^ (n = 127, N = 2)*	419 ± 10	251 ± 5	1.69 ± 0.03	0.035 ± 0.003	11.0 ± 0.5
Bassoon *RIM-BP2^−/−^(n = 124, N = 2)*	420 ± 10	249 ± 5	1.73 ± 0.04	0.032 ± 0.003	14.0 ± 0.8
Bassoon *p*-value	0.9	0.8	0.8	0.8	0.01^*^
	Student's *t*-test	Student's *t*-test	Wilcoxon rank test	Student's *t*-test	Wilcoxon rank test

*Summary of grand average data (mean ± SEM) from 2-color STED images of immunofluorescently labeled stripe-shaped Ca_V_1.3 Ca^2+^-channel clusters or bassoon clusters (Figure 5). Fit amplitudes and full width at half maximum (FWHM) of the long and short axis of a 2D Gaussian fit function were used for quantitative analysis. Further, the ratio of long over short axis, cluster area and integral were calculated. p-values and statistical test are depicted for each dataset; significance levels*:

* *p < 0.05*,

** *p < 0.01*,

*** p < 0.001; n = numbers of AZs, N = number of mice; age of mice: p21-p23

### RIM-BP2 disruption reduced the sustained phase of exocytosis

**Figure 6:**
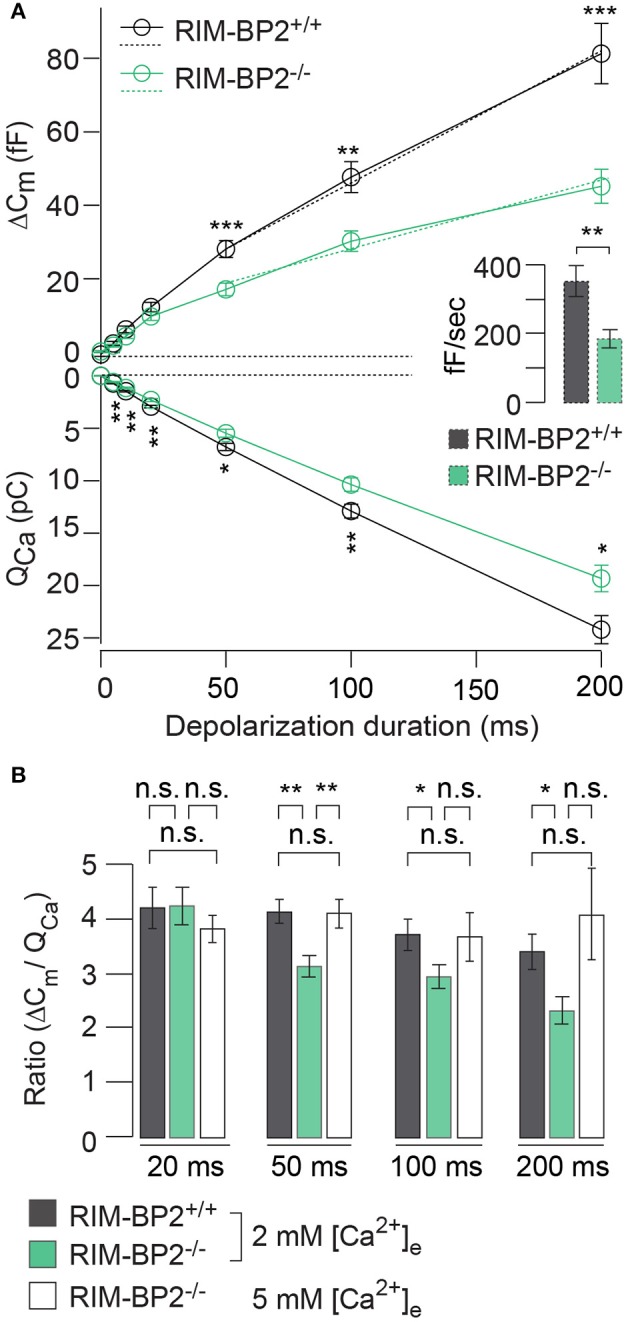
RIM-BP2 promotes sustained exocytosis in IHCs. **(A)** Left**:** Relationship of the exocytic membrane capacitance changes (Δ*C*_*m*_, top) and the corresponding whole-cell Ca^2+^-current integrals (Q_Ca_, bottom) of RIM-BP2-deficient (*RIM-BP2*^−/−^, *n* = 10, green) and control (*RIM-BP2*^+/+^, *n* = 12, black) IHCs for various depolarization durations to −14 mV. Ca^2+^-current integrals were significantly reduced during all depolarization durations (*p* = 0.01–0.002). Exocytic Δ*C*_*m*_ was significantly reduced in *RIM-BP2*^−/−^ IHCs during 50 (*p* = 0.0007), 100 (*p* = 0.002), and 200 ms (*p* = 0.0007) depolarizations representing the sustained phase of SV release whose rate is governed by vesicle resupply to the RRP. A line was fit to the sustained phase of exocytosis to each individual IHC (from 50 to 200 ms, dashed line) to estimate the kinetics of vesicle turnover during ongoing stimulation. Right: IHC grand average (mean ± SEM) of line fit slopes as estimates for SV turnover rates. A significant reduction (Wilcoxon rank test, *p* = 0.001) was observed in *RIM-BP2*^−/−^ IHCs (*n* = 10, green) compared to control *RIM-BP2*^+/+^ (*n* = 12, black) IHCs. **(B)** The ratio the exocytic Δ*C*_*m*_ and its corresponding integrated Ca^2+^-current (Q_Ca_) showed a significant difference between the two genotypes for the sustained phase of exocytosis (50–200 ms, *p* = 0.01–0.003), but not for RRP exocytosis (20 ms, *p* = 0.9). After the extracellular [Ca^2+^]_e_ was elevated from 2 to 5 mM in *RIM-BP2*^−/−^ (*n* = 7) IHCs the ratio between Δ*C*_*m*_ and Q_Ca_ was indistinguishable from *RIM-BP2*^+/+^ levels (*p* = 0.4–0.9). **(A,B)** Data information: Data represent IHC grand averages, mean ± SEM; Student's *t*-test unless specified differently (see above), Significance levels: n.s. *p* ≥ 0.05, ^*^p < 0.05, ^**^p < 0.01, ^***^p < 0.001; *n* = number of IHCs; age of mice: p14-p16.

RIM-BP2 has been indicated to serve as molecular link between Ca^2+^-channels and release ready SVs via Rab3/RIMs interaction (Acuna et al., 2015; Grauel et al., 2016). Hence, next we investigated the role of RIM-BP2 in regulating synaptic transmission either via its promotion of Ca^2+^-channel abundance, coupling Ca^2+^-channels and vesicular release sites or by directly affecting SV exocytosis. We addressed this possibility by perforated patch-clamp recordings of exocytic membrane capacitance changes (Δ*C*_*m*_) in response to voltage-gated Ca^2+^-influx in *RIM-BP2*^+/+^ and *RIM-BP2*^−/−^ IHCs. We studied RRP and sustained exocytosis by probing Δ*C*_*m*_ in response to depolarizations of various durations to the potential eliciting the maximum Ca^2+^-current (−14 mV, Figure 6A). RRP exocytosis, approximated as Δ*C*_*m*_ evoked by 20 ms depolarizations (Moser and Beutner, 2000), tended to be smaller in *RIM-BP2*^−/−^ IHCs (reduced by 21%, but not reaching statistical significance: *p* = 0.1). The sustained phase of SV exocytosis, probed by responses to stimuli longer than 20 ms, is thought to primarily reflect vesicle resupply to the RRP and subsequent fusion (Schnee et al., 2005; Goutman and Glowatzki, 2007; Meyer et al., 2009; Neef et al., 2009). The reduction in sustained exocytosis was more pronounced (e.g., 38% reduction of Δ*C*_*m*_ for 50 ms, *p* = 0.0007). In addition, a line was fit to the sustained phase of exocytosis (from 50 to 200 ms) to estimate the kinetics of SV exocytosis during ongoing depolarization (Figure 6A, dashed lines in top left panel). In *RIM-BP2*^−/−^ IHCs, SV exocytosis rates were significantly slower (193 fF/s) compared to *RIM-BP2*^+/+^ IHCs (279 fF/s, *p* = 0.001, Figure 6A, inset).

Next, we tested, whether relating the exocytic Δ*C*_*m*_ to the corresponding integrated Ca^2+^-current Q_Ca_ (Figure 6B) reduced the discrepancy of the exocytic responses between the *RIM-BP2*^+/+^ and *RIM-BP2*^−/−^ IHCs. Indeed, the Δ*C*_*m*_/Q_Ca_ ratio was indistinguishable between both genotypes for 20 ms depolarizations (*p* = 0.9). However, we still observed a significant difference between the two genotypes regarding the sustained phase of exocytosis (Δ*C*_*m*_/Q_Ca_ for 50 ms: *p* = 0.003, for 100 ms: *p* = 0.04, for 200 ms: *p* = 0.01). This indicated that the reduction of presynaptic Ca^2+^-influx does not fully account for the impaired sustained exocytosis, suggesting a role of RIM-BP2 in SV-replenishment. When raising the extracellular Ca^2+^ concentration ([Ca^2+^]_e_) from 2 to 5 mM, thereby boosting single channel Ca^2+^-current, we observed a rescue of the exocytic response of *RIM-BP2*^−/−^ IHCs that then was indistinguishable from *RIM-BP2*^+/+^ at 2 mM [Ca^2+^]_e_ (50 ms *p* = 0.9, 100 ms: *p* = 0.9, 200 ms: *p* = 0.4) (Figure 6B). This suggests that boosting single Ca^2+^-channel current can overcome the alteration imposed by RIM-BP2 disruption, potentially because Ca^2+^-domains around open Ca^2+^-channels spread further out reaching more remotely positioned fusion-competent vesicles.

### Loss of RIM-BP2 does not change the apparent Ca^2+^-cooperativity of RRP exocytosis

**Figure 7:**
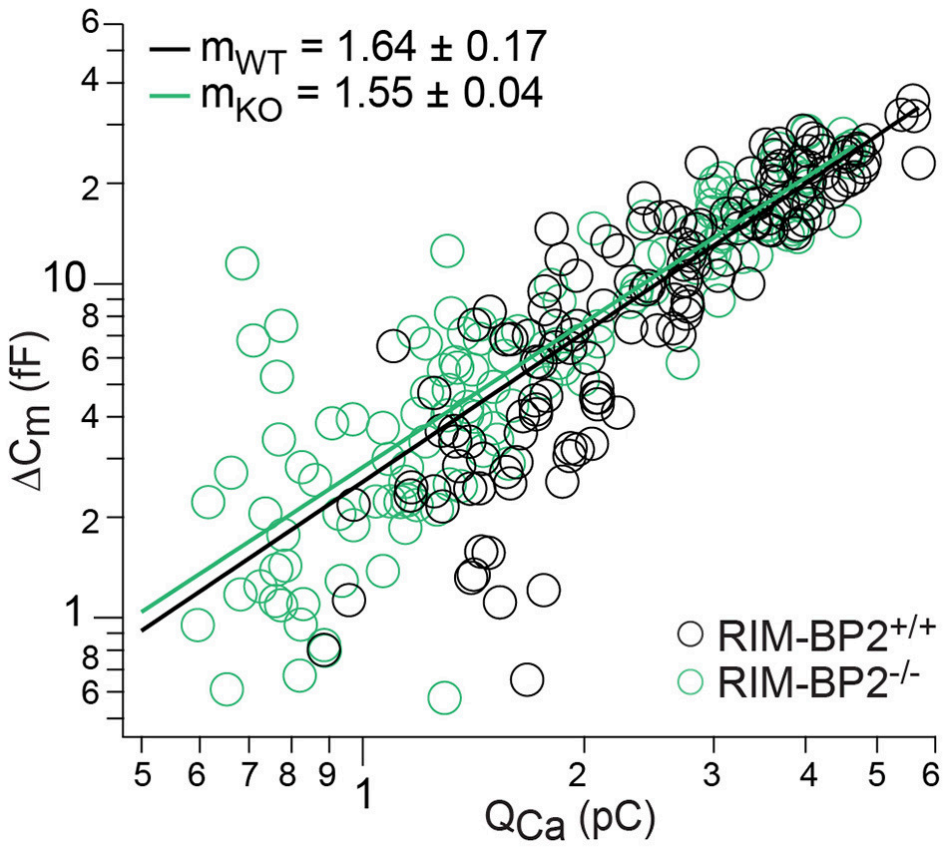
RIM-BP2 disruption did not affect Ca^2+^-influx—exocytosis coupling. Double-logarithmic plot of exocytic membrane capacitance changes (Δ*C*_*m*_) and corresponding whole-cell Ca^2+^-current integrals (Q_Ca_) during 20 ms depolarizations and perfusion of IHCs with isradipine to gradually decrease the Ca^2+^-influx exocytosis. Symbols represent individual data points from RIM-BP2-deficient (*RIM-BP2*^−/−^, *n* = 7, each 20–43 data points, green) and control (*RIM-BP2*^+/+^, *n* = 6, each 20–42 data points, black) IHC depolarizations. Solid lines represent best fit power function [Δ*C*_*m*_ = A(Q_Ca_)^m^] to all data points of each genotype. The estimates of the apparent Ca^2+^-cooperativity *m* for each individual cell were statistically indistinguishable (*p* = 0.5) between genotypes indicating normal Ca^2+^-influx—exocytosis coupling; Data information: Student's *t*-test, *n* = number of IHCs; age of mice: p14-p16.

Next, we studied Ca^2+^-influx–exocytosis coupling by probing the apparent Ca^2+^-cooperativity *m* of RRP exocytosis (Augustine et al., 1991). We had previously found *m* to be close to unity for IHCs after the onset of hearing, indicating a Ca^2+^-nanodomain-like control of exocytosis in IHCs (Brandt et al., 2005; Wong et al., 2014; Pangršič et al., 2015). If RIM-BP2 was a molecular linker between the Ca_V_1.3 Ca^2+^-channel complex and the vesicular release site, essential for establishing this Ca^2+^-nanodomain-like control, we would expect an increase in *m*. We repetitively applied 20 ms depolarizations separated by at least 60 s for SV-replenishment to secure complete RRP recovery and successively reduced the number of open Ca^2+^-channels by slow perfusion of the dihydropyridine channel L-type Ca^2+^-channel antagonist isradipine (10 μM, Figure 7). We obtained *m* by fitting the relationship of Δ*C*_*m*__,20 ms_ and corresponding Q_Ca_ for each cell with a power function Δ*C*_*m*_ = A(Q_Ca_)^*m*^ restricted to the range that did not show obvious saturation of Δ*C*_*m*__,20 ms_ (Figure 7). The estimates of *m* were statistically indistinguishable between *RIM-BP2*^−/−^ IHCs and *RIM-BP2*^+/+^ IHCs (1.55 ± 0.04 vs. 1.64 ± 0.17, *p* = 0.5) arguing for intact Ca^2+^-influx–exocytosis coupling for RRP SVs, at least after full RRP recovery. This, however, does not rule out a function of RIMB-BP2 in promoting the engagement of newcoming vesicles with Ca^2+^-channels. In fact, the reduction of sustained exocytosis as well as its increased Ca^2+^-dependence would be in line with such a scenario.

### RIM-BP2 contributes to fast synaptic vesicle recruitment

**Figure 8:**
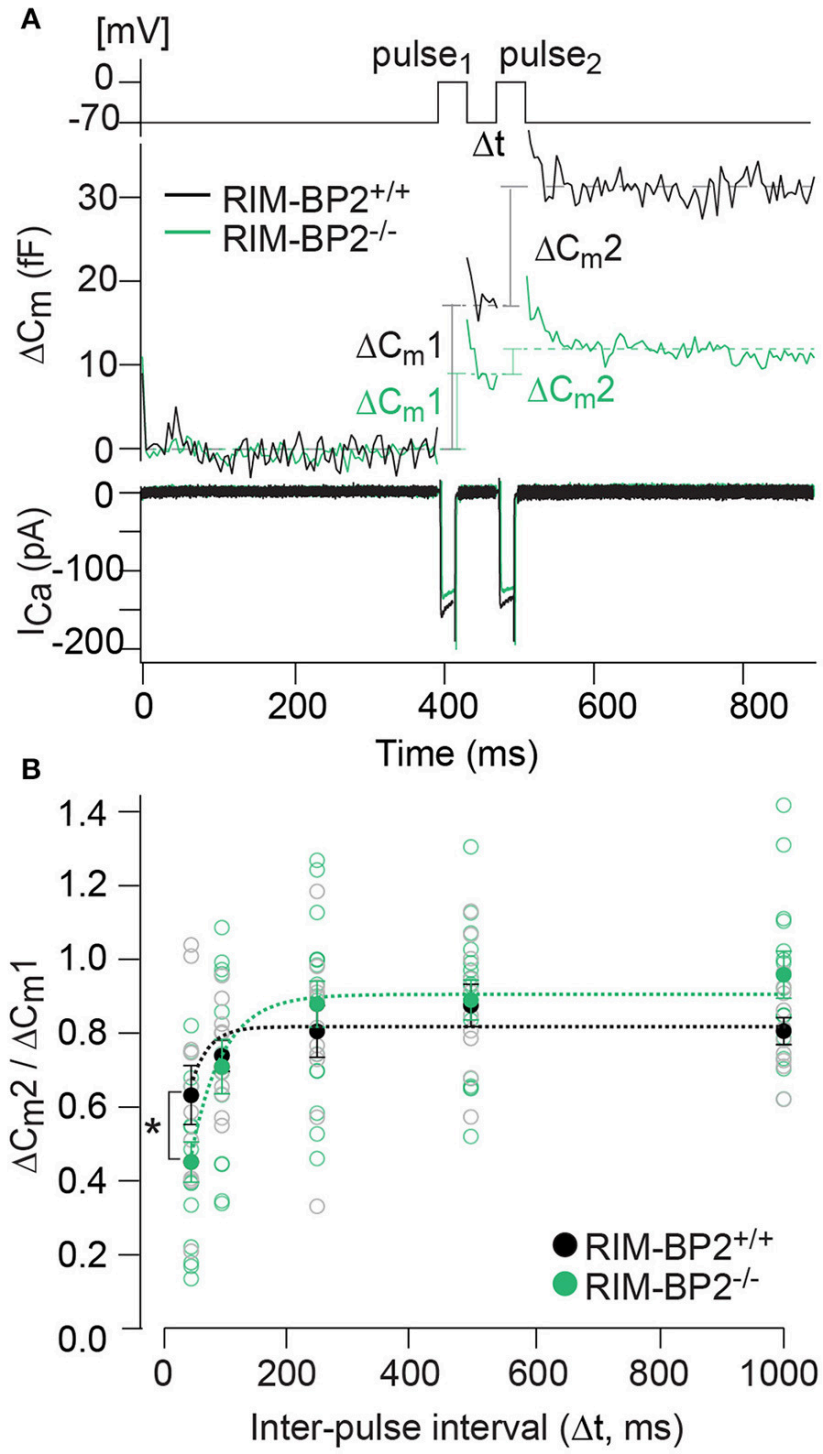
RIM-BP2 facilitates fast recruitment of SVs after RRP-depletion. **(A)** A paired-pulse protocol was used to measure SV replenishment after RRP-depletion. Average exocytic membrane capacitance traces (*C*_*m*_, middle) and corresponding Ca^2+^-currents (I_Ca_, bottom) of *RIM-BP2*^−/−^ (*n* = 18, green) and control (*RIM-BP2*^+/+^*, n* = 14, black) IHCs in response to a pair of 20 ms depolarizations separated by an inter-pulse interval (Δt) of 50 ms (top). SV replenishment was measured as the paired-pulse ratio between the second and the first exocytic membrane capacitance changes (Δ*C*_*m*_2/Δ*C*_*m*_1). **(B)** Paired-pulse ratios (Δ*C*_*m*_2/Δ*C*_*m*_1) at varying inter-pulse intervals (Δt) of RIM-BP2-deficient (*RIM-BP2*^−/−^*, n* = 18, green) and control (*RIM-BP2*^+/+^, *n* = 14, black) IHCs were significantly reduced in *RIM-BP2*^−/−^ IHCs for RRP recovery times of 50 ms (*p* = 0.02). For longer RRP recovery times (>50 ms) RRP replenishment seemed normal in RIM-BP2-deficient IHCs (*p* = 0.1–0.8). The time course of RRP recovery was estimated by fitting the paired-pulse data with a single exponential fit function (dashed lines). τ was significantly slower in *RIM-BP2*^−/−^ IHCs (*RIM-BP2*^−/−^, *n* = 5, green) compared to control (*RIM-BP2*^+/+^, *n* = 5, black) IHCs (*p* = 0.04). Data represent IHC averages (empty circles) and IHC grand averages, mean ± SEM (filled circles); Student's *t-*test: ^*^p < 0.05. **(A,B)** *n* = number of IHCs; age of mice: p14-p16.

Our next step was to test for a role of RIM-BP2 in replenishing SVs during RRP recovery from depletion using a paired-pulse paradigm. We used a 20 ms depolarization to −14 mV to deplete the RRP and then probed its recovery by a consecutive 20 ms pulse applied within variable intervals (Δt) to the first pulse (Figure 8A). RRP recovery was slowed in *RIM-BP2*^−/−^ IHCs as evident from significantly smaller responses at an interval of 50 ms (*p* = 0.02, Figure 8B). The time course of RRP recovery was estimated by fitting the paired-pulse data with a single exponential function (Figure 8B, dashed lines) where the RRP refilling time constant amounted to 51 ms in *RIM-BP2*^−/−^ IHCs compared to 27 ms in *RIM-BP2*^+/+^ IHCs. These two data sets indicate that in IHC ribbon synapses, RIM-BP2 is required for fast recruitment of new incoming SVs to the release sites after RRP-depletion. We speculate that RIM-BP2 could help to quickly guide SVs into close proximity of a nearby Ca^2+^-channel. Longer RRP recovery times (>50 ms) were sufficient for normal RRP replenishment in RIM-BP2-deficient IHCs (Figure 8B). Likely, longer recovery times increase the probability of proper positioning of replenished SVs to their release site by alternative protein-protein interactions.

### Loss of RIM-BP2 changes the synaptic vesicle distribution at the presynaptic membrane

**Figure 9:**
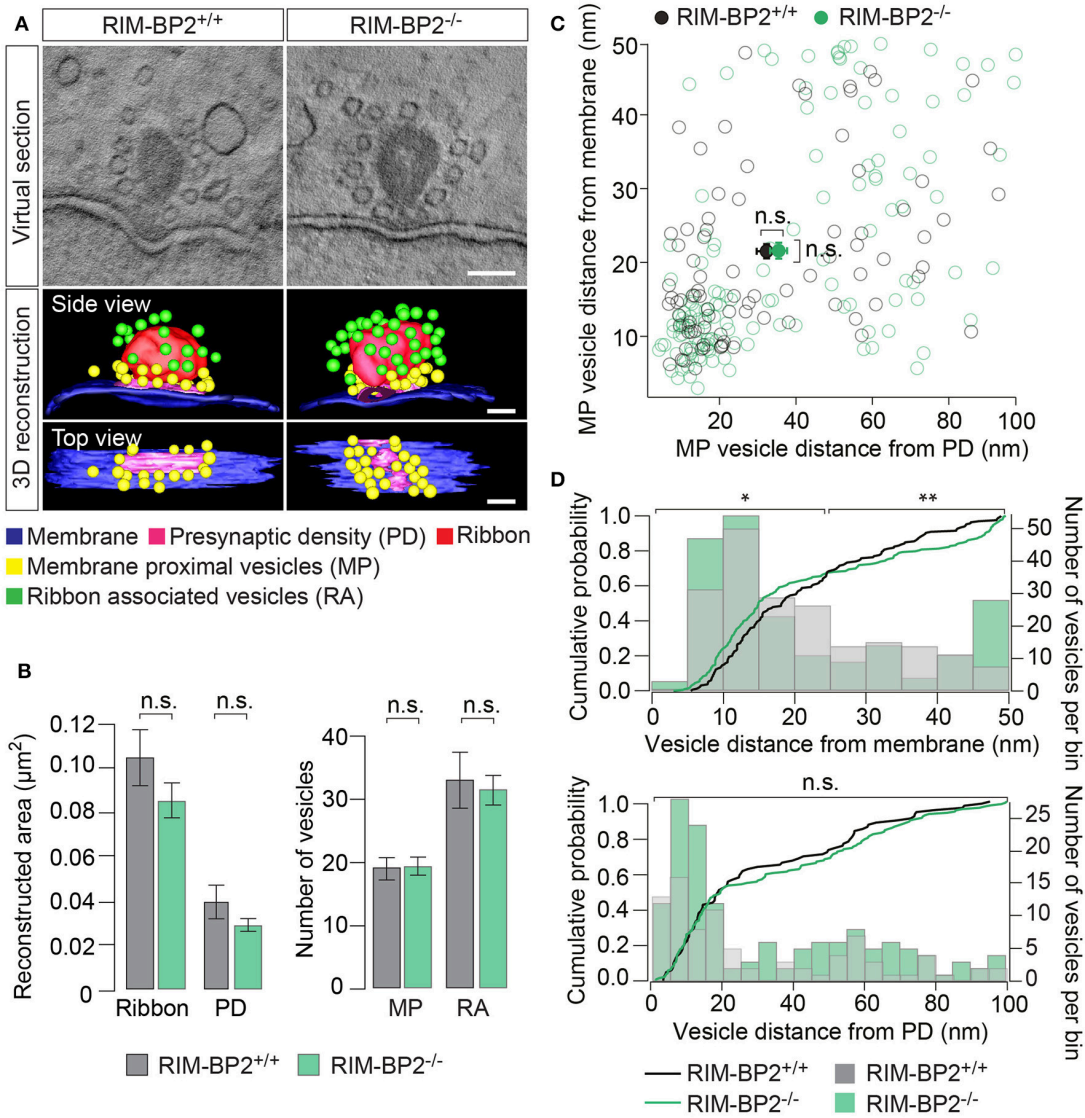
RIM-BP2 regulates the distance of SVs to the presynaptic membrane. **(A)** Exemplary virtual sections of *RIM-BP2*^−/−^ and *RIM-B2*^+/+^ ribbon synapses (Top) and side and top view of respective 3D models (Bottom). The overall synapse ultrastructure in *RIM-BP2*^−/−^ IHCs was normal: Synaptic ribbons (red) were anchored to the presynaptic membrane (blue) via a presynaptic density (PD, pink). SVs that were located within a 100 nm distance from the PD and with a membrane-to-membrane distance of ≤50 nm away from the presynaptic plasma membrane were classified as “membrane proximal” SVs (MP-SVs, yellow). SVs that appeared within 80 nm around the presynaptic ribbon within the first SV layer around the ribbon were considered as “ribbon associated” SVs (RA-SVs, green). Scale bar: 100 nm **(B)** Quantitative analysis of electron tomograms: reconstructed ribbon (Student's *t*-test *p* = 0.13) and PD area (Wilcoxon rank test *p* = 0.13) (left) as well as the average number of MP (Student's *t*-test *p* = 0.14) and RA (Student's *t*-test *p* = 0.76) SVs (right) were unaltered in *RIM-BP2*^−/−^ AZs (*n* = 9, *N* = 2) compared to *RIM-BP2*^+/+^ AZs (*n* = 9, *N* = 2). Data represent grand averages, mean ± SEM; Significance level: n.s. *p* ≥ 0.05; *n* = number of tomograms, *N* = number of mice; age of mice: p21 **(C)** shortest (membrane-to-membrane) distance of MP-SVs [from B] to the plasma membrane and presynaptic density. Plot shows distances of individual SVs (open circles) and mean ± SEM (closed circles) in *RIM-BP2*^−/−^ AZs (*n* = 9, *N* = 2) and *RIM-BP2*^+/+^ AZs (*n* = 9, *N* = 2) with no significant difference regarding the average distance of MP SVs to the membrane (Student's *t*-test *p* = 0.8) or the PD (Wilcoxon rank test, *p* = 0.6). **(D)** Distribution of MP-SVs **(B)** regarding their shortest (membrane-to-membrane) distance to the plasma membrane (Top) and the PD (Bottom). Histograms show the number of SVs (bin size 5 nm, right y-axis) with respect to their distance to the plasma membrane (Top) or PD (Bottom). The cumulative density function (solid line) shows the probability of SVs (left y-axis) being located at a specific distance to the membrane (Top) or the PD (Bottom). The Kolmogorov-Smirnov test was used to compare the probability distribution of SVs. Top: A large fraction (70%) of SVs was located within ~25 nm and a small fraction (30%) of SVs was located within ~25–50 nm away from the plasma membrane. A significantly different distribution of SVs was observed for both the large (*p* = 0.004) and the small (*p* = 0.02) fractions of MP-SVs. Bottom: No difference in the distribution of SVs was observed with respect to the PD (*p* = 0.7). Comparing the distribution of SVs from the small and large pool as defined for the plasma membrane distribution, but with respect to the PD, also revealed no difference in the SV distribution with respect to the PD (small fraction: *p* = 0.6, large fraction *p* = 0.07). **(C,D)** Significance levels: n.s. *p* ≥ 0.05, ^*^p < 0.05, ^**^p < 0.01.

To examine the effect of RIM-BP2 disruption on an ultrastructural level, we studied IHC ribbon synapses in RIM-BP2^−/−^ and *RIM-BP2*^+/+^ mice using electron tomography (Figure 9). The overall synapse ultrastructure appeared normal: the size of synaptic ribbons was unaltered (Figure 9B) and synaptic ribbons were anchored to the presynaptic membrane via the presynaptic density (PD) (Figure 9A), which was normal in size as well (Figure 9B). Further, the number of SVs directly facing the AZ membrane (membrane-proximal SVs, MP-SVs, see section Materials and Methods) and of SVs directly adjacent to the ribbon (ribbon-associated SVs, RA-SVs) was unaltered (Figure 9B).

Next, we analyzed the distribution of MP-SVs regarding their distance to the plasma membrane and PD (Figures 9C,D). On average, no difference in the MP-SV distance to the membrane or PD was observed (Figure 9C). The cumulative distribution function of membrane-to-membrane distance however, shows that a large fraction (70%) of MP-SVs was located within ~25 nm from the plasma membrane and a smaller fraction (30%) of all MP-SVs between ~25 and 50 nm at AZs of both genotypes (Figure 9D top). Interestingly, the distributions differed for both fractions of MP-SVs between *RIM-BP2*^+/+^ and *RIM-BP2*^−/−^ AZs (small fraction: *p* = 0.02 and large fraction: *p* = 0.004). There was a tendency of more MP-SVs in *RIM-BP2*^−/−^ AZs to populate the smaller distance bins (Figure 9D), while we observed less SVs with intermediate distance (~20–40 nm), but a second accumulation of MP-SVs further away from the membrane (~40–50 nm) that seemed absent at *RIM-BP2*^+/+^ AZs. The lateral distribution of MP-SVs with respect to the PD was unaltered in *RIM-BP2*^−/−^ AZs (Figure 9D bottom).

Presence and unaltered size of ribbon and PD are consistent with our above immunofluorescence analysis (Figures 1, 2, 4). The normal count of MP-SVs suggests that exocytosis is not limited by the availability of SVs. We speculate that RIM-BP2 might instead promote fast replenishment of readily releasable vesicles by rapidly engaging them with Ca_V_1.3 channels, i.e., by positional priming (Neher and Sakaba, 2008), that we might not have resolved by our static tomographic analysis.

### Disruption of RIM-BP2 causes a mild impairment of synaptic sound encoding

**Figure 10:**
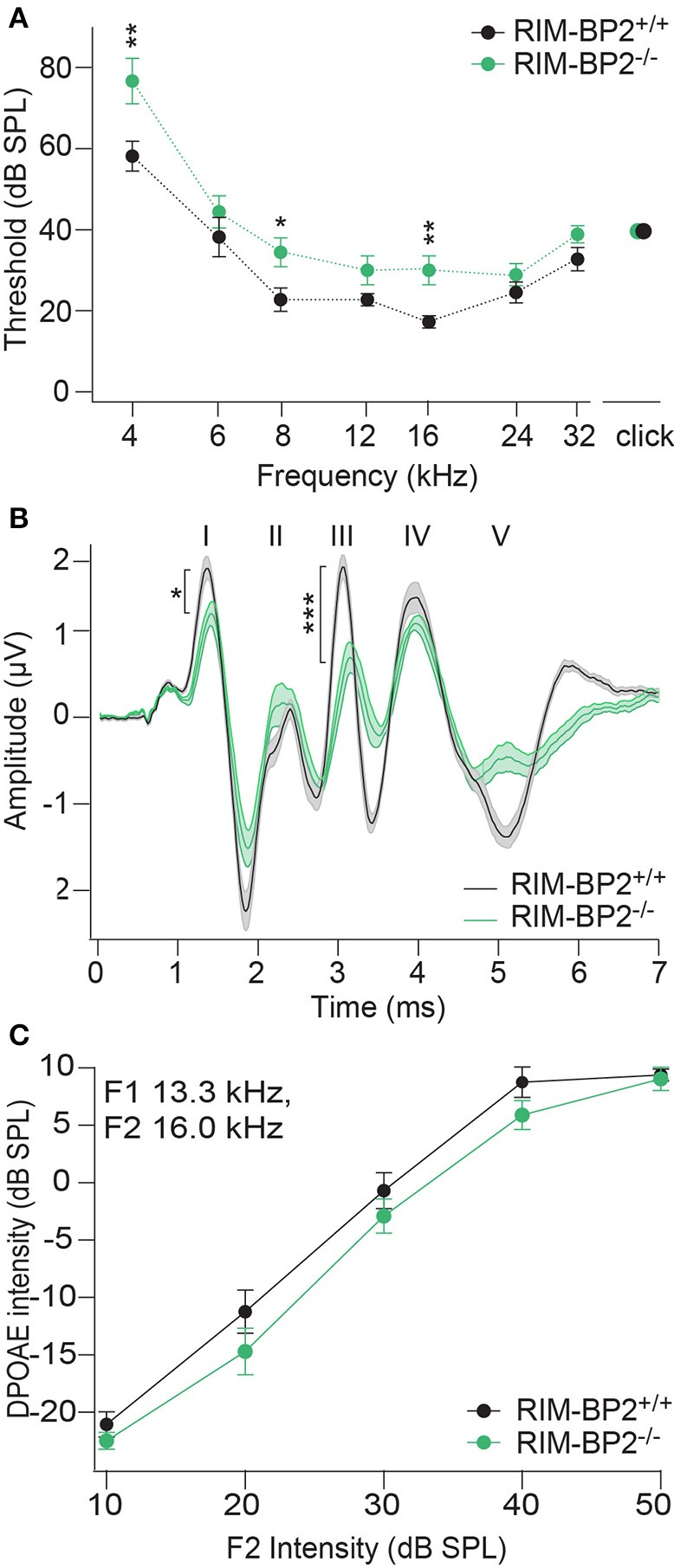
RIM-BP2 disruption caused a mild synaptopathic hearing impairment. **(A)** Auditory brainstem response (ABR) thresholds were elevated in RIM-BP2-deficient mice compared to control mice especially at 4 (*p* = 0.005), 8 (*p* = 0.02), and 16 kHz (*p* = 0.002). **(B)** Compared to control mice ABR waveforms (80 dB peak equivalent, 20 Hz stimulation rate) of RIM-BP2-deficient mice elicited a significantly reduced ABR wave I amplitude (*p* = 0.03), representing the compound action potential of SGNs. **(C)** At the frequency of strongest hearing threshold increase 16 kHz, see **(A)**, otoacoustic emission amplitudes were unaltered in RIM-BP2-deficient mice compared to control mice indicating normal mechanoelectrical transduction and cochlear amplification (F1: 13.3 kHz, F2: 16 kHz) (*p* = 0.1–0.9). **(A–C)** *RIM-BP2*^+/+^ (*n* = 11), *RIM-BP2*^−/−^ (*n* = 8); Data information: Data represent grant averages, mean ± SEM; one way ANOVA, *p*-values are from post hoc Tukey's multiple comparison: ^*^p < 0.05, ^**^p < 0.01, ^***^p < 0.001; *n* = number of mice; age of mice: 8–10 weeks.

In order to probe the impact of RIM-BP2 on auditory systems function we first recorded auditory brainstem responses (ABR) and distortion product otoacoustic emissions (DPOAE) in *RIM-BP2*^−/−^mice. While DPOAE test the function of the cochlear amplification mediated by outer hair cells, ABR reflect the synchronized neural activation of the early stages of the auditory pathway. ABR thresholds were mildly but significantly elevated in *RIM-BP2*^−/−^ mice (Figure 10A; *p*_4kHz_ = 0.005, *p*_8kHz_ = 0.02, *p*_16kHz_ = 0.002). In addition, ABR wave I amplitude, reporting the compound action potential of the SGNs, was significantly reduced (Figure 10B; *p* = 0.03). As the presence of otoacoustic emissions with normal amplitudes (for all F2 intensities: *p* > 0.05) (Figure 10C) indicates normal mechanoelectrical transduction and cochlear amplification, this suggests a mild synaptopathic hearing impairment (Moser and Starr, 2016). Potentially, the hearing phenotype might still be attenuated by the presence of RIM-BP1 or −3 in IHCs, which should be tested in future studies.

**Figure 11:**
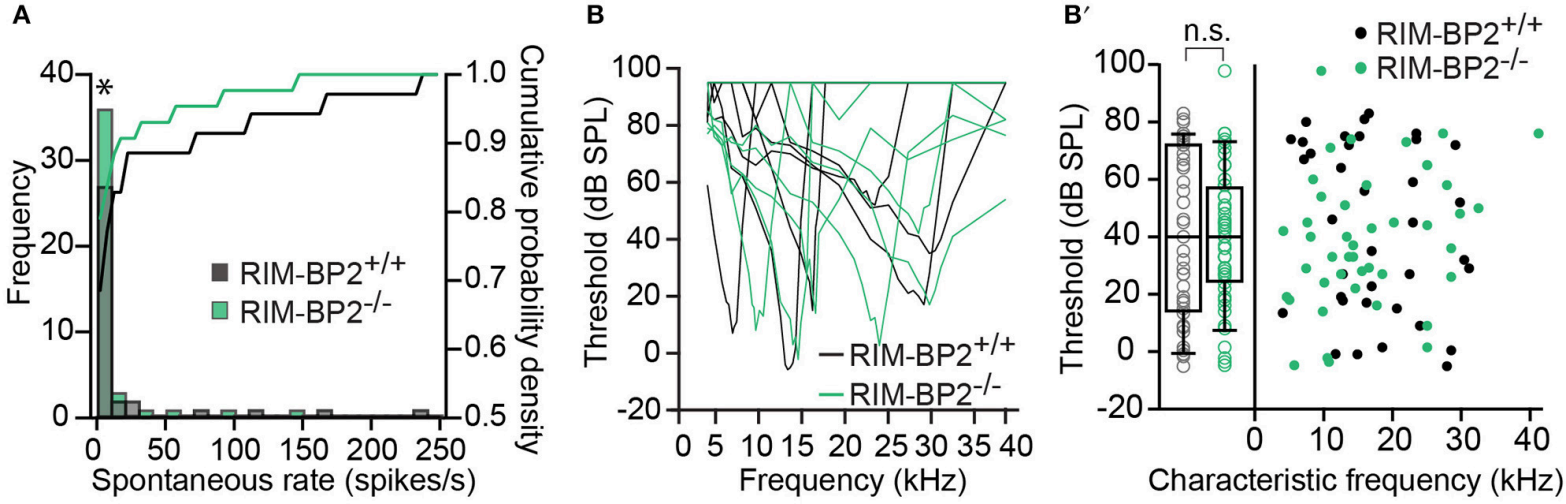
Higher fraction of fiber with low spontaneous firing rates in single unit recordings from *RIM-BP2*^−/−^ auditory nerve fibers **(A)** Distribution of spontaneous firing rates of single units in *RIM-BP2*^+/+^ (black; *n* = 35; *N* = 6) and *RIM-BP2*^−/−^ (green; *n* = 43; *N* = 3) auditory nerve fibers (ANFs). The histogram represents the distribution of their frequency (left y-axis) and the solid lines represent the cumulative probability density (right y-axis) of spontaneous firing rates. The fraction of ANFs with low spontaneous firing rates was significantly higher in *RIM-BP2*^−/−^ mice. Wilcoxon rank test, *p* = 0.04. **(B)** Representative tuning curves of ANF from *RIM-BP2*^+/+^ (black; *n* = 36; *N* = 6) and *RIM-BP2*^−/−^ (green; *n* = 43; *N* = 4) mice demonstrate easily distinguishable characteristic frequencies. **(B**′**)** ANF sound thresholds at their characteristic frequency were comparable between the two genotypes: 41.68 ± 5.03 dB for *RIM-BP2*^+/+^ (*n* = 36; N = 6) and 38.42 ± 3.6 dB for *RIM-BP2*^−/−^ (*n* = 43; *N* = 4). Student's *t*-test, *p* = 0.60. Each data point represents the response of single unit of ANF. **(A,B)** Data information: significance levels: n.s. *p* ≥ 0.05, ^*^p < 0.05; *n* = number of single units of ANFs and *N* = number of mice. Box and whisker plot represents median, lower/upper quartiles and 10th−90th percentiles.

**Figure 12:**
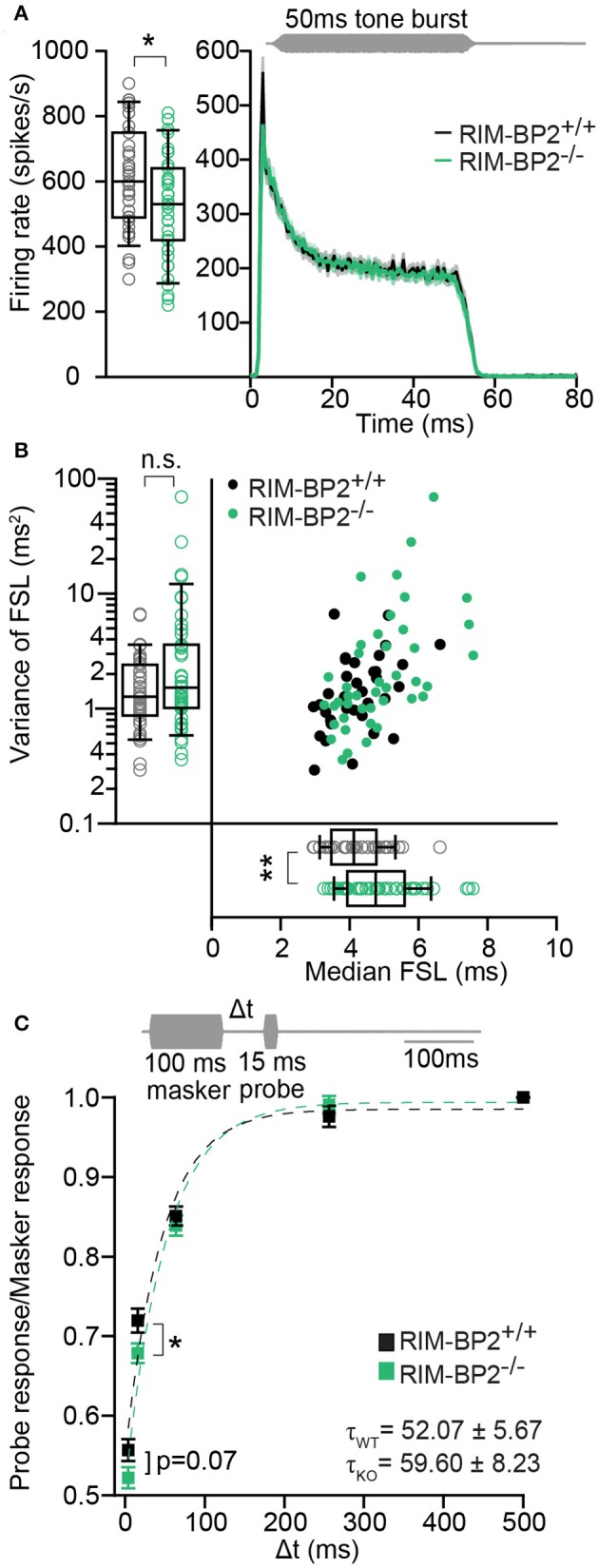
*RIM-BP2*^−/−^ auditory nerve fibers have reduced onset firing rates, an increased postsynaptic first spike latency and slower recovery. **(A)** Peak-aligned peri-stimulus time histogram (PSTH) of the response of ANFs to 50 ms tone burst stimulation (at characteristic/best frequency, 30 dB above threshold; stimulus paradigm illustrated in gray) in *RIM-BP2*^+/+^ (black; *n* = 35; *N* = 6) and *RIM-BP2*^−/−^ (green; KO *n* = 43; *N* = 4). PSTH presented as mean (solid lines) ± SEM (shaded area). Peak onset firing rate was reduced in *RIM-BP2*^−/−^ ANFs. Student's *t*-test, *p* = 0.02. **(B)** Median first spike latency of PSTH **(A)** was increased in *RIM-BP2*^−/−^ ANFs (Student's *t*-test, *p* = 0.004) while the variance in first spike latency remained unperturbed. Wilcoxon rank test, *p* = 0.23. Each data point represents the response of single unit of ANF. **(C)** The time course of recovery of the ANF was assessed by a 100 ms masker tone followed by 15 ms probe tone presented after a silent interval (recovery time) of variable duration (Δt) (at characteristic/best frequency, 30 dB above threshold; stimulus paradigm illustrated in gray). Recovery was plotted as the ratio of ANF peak response to probe tone to the ANF peak response to masker tone (solid boxes ± SEM). Student's *t*-test, *p* = 0.04. Dotted lines represent single exponential fits to the time course of recovery. Time constants (τ) of recovery, displayed on the graph as mean ± SEM were comparable between the two genotypes (Wilcoxon rank test, *p* = 0.81). *RIM-BP2*^+/+^ (black; *n* = 40; *N* = 6) and *RIM-BP2*^−/−^ (green; *n* = 43; *N* = 4). **(A–C)** Data information: Significance levels: n.s. *p* ≥ 0.05, ^*^p < 0.05, ^**^p < 0.01; *n* = number of single units of ANFs and *N* = number of mice. Age of mice: 9–10 weeks. Box and whisker plot represents median, lower/upper quartiles and 10th−90th percentiles.

Using extracellular recordings we studied sound encoding by single postsynaptic SGNs, which offers *in vivo* analysis of single AZ function as each SGN is thought to receive input from just one AZ of one IHC (Liberman, 1978). We stereotactically targeted microelectrodes to where the auditory nerve fibers (central axons of SGNs) enter the AVCN and recorded spontaneous and sound evoked firing in neurons that most likely represent SGNs (“putative” SGNs, hereafter dubbed SGNs for simplicity, see section Materials and Methods). We observed a larger proportion of SGNs exhibiting low spontaneous firing in *RIM-BP2*^−/−^ mice (Figure 11A; *p* = 0.04), which might result from the reduction in the Ca^2+^-channel abundance at the IHC ribbon synapses (Figures 4, 5). We found normal frequency tuning (Figure 11B) and sound thresholds (Figure 11B′) of *RIM-BP2*^−/−^ SGNs, which supports our above notion of intact cochlear amplification (Figure 10C). We then studied the neural response to 50 ms tone bursts at the characteristic frequency at a sound pressure level of 30 dB above threshold (Figure 12A), which is considered to reflect the presynaptic RRP dynamics convolved with postsynaptic refractoriness (Wittig and Parsons, 2008; Buran et al., 2010; Frank et al., 2010). The peak firing rate reports the maximal initial rate of SV release from the SV-occupied release sites of the standing RRP, (Pangršič et al., 2012). The partial depletion of the RRP governs the rapid spike rate adaptation and the adapted firing rate reflects the balance of SV fusion and replenishment. We found a reduced peak firing rate in *RIM-BP2*^−/−^ SGNs (*p* = 0.02), while the adapted firing rates where indistinguishable between SGNs of both genotypes (Figure 12A). The first spike latency was significantly delayed in *RIM-BP2*^−/−^ SGNs (*p* = 0.004), but the trend toward an increased variance of first spike latency did not reach statistical significance (Figure 12B). Together, these findings indicate a mild impairment of sound onset coding, which likely results in less synchronized firing of the SGN population thereby explaining the ABR phenotype. Sound coding during continued stimulation, therefore also SV-replenishment, on the other hand, seemed unaltered, which contrasts our results on IHC physiology (Figure 8). Therefore, we tested SV-replenishment as recovery on sound onset coding at variable times after cessation of stimulation (forward masking paradigm (Harris and Dallos, 1979), which provides an estimate of the kinetics of the RRP recovery after partial depletion (Figure 12C). Probe responses at short intervals to a 100 ms masker tone tended to be smaller for *RIM-BP2*^−/−^ SGNs for 4 ms (*p* = 0.07) and significantly so for 16 ms (*p* = 0.04), but from the interval of 64 ms onwards, the recovered response was comparable to that of *RIM-BP2*^+/+^ SGNs, and the time constant for recovery from masking was unaltered. In summary, recordings from single SGNs indicated a mild impairment of sound onset coding and suggested that fast vesicle replenishment is slowed in the absence of RIM-BP2.

## Discussion

In this study, we examined the expression of RIM-BP2 in mouse IHCs and studied the structural and functional effects of genetic RIM-BP2-deletion in IHC ribbon synapses. We demonstrate the presence of RIM-BP2 at the IHC AZs where it positively regulates (1) the number of synaptic Ca^2+^-channels and, in addition, (2) promotes fast SV-replenishment. STED microscopy indicated that RIM-BP2 is part of the typically stripe-like appearing presynaptic density at IHC AZs. Recordings of whole-cell and synaptic Ca^2+^-influx as well as STED microscopy of Ca_V_1.3 immunofluorescence collectively demonstrated a reduced abundance of Ca_V_1.3 Ca^2+^-channels at IHC AZs upon disruption of RIM-BP2. Sustained exocytosis was reduced. RRP exocytosis and its Ca^2+^-nanodomain-like control by Ca^2+^-channels seemed preserved when probed with long inter-stimulus intervals. RRP replenishment, however, was slowed in RIM-BP2-deficient IHCs. In addition, subtle morphological changes in the spatial organization of SVs at the plasma membrane were found by electron tomography. Finally, we found a mild impairment of sound onset coding in SGNs of *RIM-BP2*^−/−^ mice that likely reflects the presynaptic impairment of Ca^2+^ signaling and SV-replenishment. In summary, we propose that RIM-BP2 promotes the clustering of Ca_V_1.3 channels at the IHC AZs and supports fast RRP replenishment.

### Molecular regulation of presynaptic Ca^2+^-channels at inner hair cell active zones

Sensory hair cells provide great access for biophysical analysis of Ca^2+^-influx and exocytosis enabling presynaptic studies of Ca^2+^-channels and their regulation of transmitter release. Ribbon-type AZs of IHCs, on average, are thought to cluster ~80–100 Ca^2+^-channels (Brandt et al., 2005; Zampini et al., 2013), which is very similar to frog saccular AZs (90 Ca^2+^-channels) (Roberts et al., 1990). So far, a positive regulation of the number and nanoscale organization of Ca_V_1.3 Ca^2+^-channels was shown for the scaffold proteins bassoon and RIM. RIMs, intensively studied interacting partners of Ca_V_ channels (Coppola et al., 2001; Kiyonaka et al., 2007; Gebhart et al., 2010; Kaeser et al., 2011; Picher et al., 2017), promote clustering of Ca_V_1.3 Ca^2+^-channels at the AZs of IHCs, but stripe-like Ca^2+^-channels clusters are maintained despite RIM2α disruption (Jung et al., 2015). Disruption of bassoon, on the other hand, not only led to a major reduction in the number of synaptic Ca^2+^-channels, but in addition caused a disintegration of the Ca^2+^-channel clusters at the IHC AZ (Frank et al., 2010). However, the interpretation of bassoon disruption is complicated as it also causes the loss of synaptic ribbons (Dick et al., 2003; Khimich et al., 2005; Jing et al., 2013). Our present study, using functional and morphological imaging in RIM-BP2-deficient IHCs indicated a ~40% reduction of the number of synaptic Ca^2+^-channels that remained clustered beneath the presynaptic density of the ribbon-occupied AZs. The decline of synaptic Ca^2+^-influx exceeded the reduction of the whole-cell Ca^2+^-influx (~20%), suggesting that AZs fail to cluster the available Ca^2+^-channels. Consistent with impaired synaptic clustering of Ca^2+^-channels we found a reduced length of the stripe-like clusters, which also showed lower integrated Ca_V_1.3 immunofluorescence intensity. Importantly, the length of the presynaptic density, measured by STED microscopy of bassoon immunofluorescence, was normal for the AZs of RIM-BP2-deficient IHCs. Together, this suggests that the reduction of Ca^2+^-channel number occurs despite the presence of the ribbon and of a normal bassoon-containing presynaptic density, placing RIM-BP2's function in channel clustering downstream of bassoon. Both, the width and area of Ca_V_1.3 clusters revealed by STED microscopy and the spread of the Ca^2+^ signal at the AZ appeared normal arguing against a mis-localization of Ca^2+^-channels to outside the area marked by the presynaptic density. Moreover, the biophysical properties of IHC Ca^2+^-channels, voltage-dependence of activation as well as extent and kinetics of inactivation, were unaltered. We cannot fully exclude changes in Ca^2+^-channel open probability due to delayed synapse maturation upon RIM-BP2 disruption. However, several findings argue against a developmental deficit in *RIM-BP*^−/−^ IHC AZs: (i) Ca^2+^-current inactivation was unaltered and mature (Grant and Fuchs, 2008), (ii) even though Ca^2+^-channel clusters were rounder, they were spatially confined and co-localize with immunolabeled presynaptic ribbons, which were present in mature numbers (Sendin et al., 2007; Zampini et al., 2010; Wong et al., 2014), (iii) finally the apparent Ca^2+^-cooperativity of exocytosis of 1.5 in *RIM-BP2*^−/−^ IHCs indicates tight Ca^2+^-nanodomain-like control of exocytosis (Wong et al., 2014). This study is the first to report a role of RIM-BPs in regulating the number of Ca^2+^-channels at the AZ of a mammalian synapse, thereby contrasting findings at hippocampal synapses and the calyx of Held synapse (Acuna et al., 2015; Grauel et al., 2016). However, a reduction in presynaptic Ca^2+^-influx was found at the neuromuscular junction of *Drosophila* larvae, which also displays an elaborate dense projection (T-bar) and requires d-RIM-BP for sufficient Ca^2+^-influx and transmitter release (Liu et al., 2011; Müller et al., 2015).

### Role of RIM-BP2 for sound encoding at the inner hair cell ribbon synapse

How does RIM-BP2 contribute to the regulation of synaptic transmission at IHC synapses that drive sound encoding in SGNs? We approached this question by analysis of IHC exocytosis, extracellular recordings from SGNs and measurements of ABR that rely on synchronous activation of SGNs. We tested two hypotheses: (i) reduction of exocytosis in proportion with the loss of presynaptic Ca^2+^-channels due to preserved Ca^2+^-nanodomain-like control of exocytosis and vesicle replenishment, (ii) reduction of exocytosis beyond that of the Ca^2+^-influx due to additional disruption of Ca^2+^-nanodomain-like control of exocytosis and/or impaired vesicle replenishment. Emerging evidence shows for neuromuscular junction of *drosophila* larvae (Liu et al., 2011; Müller et al., 2015), hippocampal synapses (Grauel et al., 2016) and the calyx of Held (Acuna et al., 2015) that genetic inactivation of RIM-BP disrupts the tight coupling between Ca^2+^-channels and release site.

Here, we tested the apparent Ca^2+^-cooperativity *m* of RRP exocytosis for changes in Ca^2+^-influx brought about by successive dihydropyridine block of Ca_V_1.3 Ca^2+^-channels. We had established by various experiments and modeling that the near linear *m* (typically around 1.5) for channel block, which contrasts an *m* of 3–4 during changes of single channel current, is an indicator for Ca^2+^-nanodomain-like control of exocytosis at the mature IHC AZ (Brandt et al., 2005; Wong et al., 2014; Pangršič et al., 2015). When testing *m* of RRP exocytosis during dihydropyridine block with inter-pulse intervals of 60 s and more, in order to secure full RRP replenishment, we found no indication for a looser Ca^2+^-influx—exocytosis coupling in RIM-BP2-deficient IHCs. The mild effect of genetic deletion RIM-BP2 on RRP exocytosis and the intact Ca^2+^-nanodomain coupling are surprising given the findings of previous studies on RIM-BP function and might suggest that IHC ribbon-type AZs can use additional molecular mechanisms to establish tight coupling of Ca^2+^-channels and release sites in the absence of RIM-BP2. In this respect it is worth mentioning, that disruption of the RIM-BP interaction partners bassoon or of RIM2α did not lead to an obvious switch to Ca^2+^-microdomain control of RRP exocytosis at IHC synapses either (Frank et al., 2010; Jung et al., 2015). It is possible that in IHCs, only combinatory deletion of several proteins like for example RIM and RIM-BP2 would elicit any measurable effect on the tight coupling of Ca^2+^-channels and release sites. Further, the observation of tight Ca^2+^-nanodomain-like control of exocytosis for fusion-competent SVs of the RRP in the absence of RIM-BP2 was made under the artificial conditions of the biophysical experiment, where the synapse was inactivated due to voltage-clamp to hyperpolarized potential for full RRP recovery. In physiology, on the other hand, IHC AZs mediate massive vesicle turnover, which requires rapid replenishment at rates of hundreds of vesicles per second (Pangršič et al., 2012). For precise sound coding under conditions of high-frequency transmission, the engagement of newcoming vesicles with one or few nearby Ca^2+^-channel complexes needs to proceed at a similar pace (Cho et al., 2011). This might require multiple mechanisms of molecular interaction posing a higher requirement for each of the candidate molecular linkers. Interestingly, we found an impairment of RRP replenishment in RIM-BP2-deficient IHCs, which seems consistent with such an increased requirement for RIM-BP2 during turnover. One conceivable interpretation would be that RIM-BP2 acts as an indirect molecular linker between SVs and Ca^2+^-channels e.g., through Rab3—RIM—RIM-BP2—Ca^2+^-channel interaction (Hibino et al., 2002; Kaeser et al., 2011). Thereby, RIM-BP2 might engage molecularly primed SVs with a nearby Ca^2+^-channel after RRP depletion. This step was found to be rate-limiting as it converts slow releasing SVs into fast releasing SVs and was called “positional priming” (Neher and Sakaba, 2008). “Positional priming” was found to be actin dependent (Lee et al., 2013), for which we did not test here. However, a recent study reported that actin secures SV replenishment in IHCs at high rates and prevents SV exhaustion during the sustained phase of exocytosis (Guillet et al., 2016). Upon Actin network disruption in IHCs, SVs, which would only be available for release after RRP depletion during longer stimulations (≥50 ms) in control condition, fused with the plasma membrane ahead of time, indicating a role of actin in preventing premature exocytosis (Guillet et al., 2016). Further, the observed shift in the MP-SV distribution with respect to the plasma membrane in *RIM-BP2*^−/−^ EM tomograms suggests a change in SV position with respect to release sites. The observed change was small, but likely, the recruitment process of SVs is so fast that it is not fully reflected in our EM data. Both paired-pulse experiments and forward masking point out a significant delay in RRP refilling for short inter-stimulus intervals, and thereby support the hypothesis that RIM-BP2 plays a role in fast RRP refilling. Additionally, the reduced number of synaptic Ca^2+^-channels in RIM-BP2 deficient AZs might enhance the delay during “positional priming,” since fewer channels are available for SVs to connect. Hence, SV turnover speed would be limited by a lower probability of SV—release site matching due to a reduced number of Ca^2+^-channels and possibly also through the slowed engagement of SVs with the remaining Ca^2+^-channels. Given the possibility of RIM-BP2 interaction with the ribbon anchor bassoon (Davydova et al., 2014) another conceivable interpretation for the slow RRP replenishment upon RIM-BP2 disruption could be a slightly increased distance between Ca^2+^-channels and the presynaptic density and thus, ribbon-tethered SV. Both, STED microscopy and Ca^2+^-imaging did not reveal changes in the width and area of Ca_V_1.3 clusters and spread of the Ca^2+^ signal, arguing against major changes in Ca^2+^-channel localization. In addition, the distance of MP SVs to the PD was unaltered in RIM-BP2 deficient synapses. However, we cannot rule out that already a slight increase of the distance of Ca^2+^-channels from the PD could slow down the engagement of new incoming ribbon-tethered SVs with a Ca^2+^-channel during RRP replenishment. Interestingly, the reduced efficiency of Ca^2+^ influx to trigger exocytosis could be rescued by elevating extracellular [Ca^2+^]_e_ in *RIM-BP2*^−/−^ IHCs. Elevated extracellular [Ca^2+^]_e_ could lead to accelerated Ca^2+^-dependent SV trafficking and replenishment which was reported for hair cells (Moser and Beutner, 2000; Spassova et al., 2004; Cho et al., 2011; Goutman and Glowatzki, 2011; Schnee et al., 2011) and other synapses (e.g., calyx of Held synapses Wang and Kaczmarek, 1998; Hosoi et al., 2007). Lower presynaptic Ca^2+^ signaling due to the reduced number of synaptic Ca^2+^-channels in RIM-BP2 deficient AZs might therefore explain their slower SV replenishment. In addition, or alternatively, increasing Ca^2+^-influx through elevation of extracellular [Ca^2+^]_e_ might recruit more distant release sites so that the exact spatial distribution and matching of Ca^2+^-channels and SVs is less critical (Fuchs et al., 2003; Pangršič et al., 2015) thereby bypassing “positional priming” as a potential rate-limiting step of exocytosis in RIM-BP2 deficient IHC synapses. A role for RIM-BP in positional priming was previously proposed based on observing slower resupply of high release probability SVs at DRBP-deficient *Drosophila* NMJ (Müller et al., 2015). Therefore, loss of RIM-BP2 likely weakens the SV replenishment at IHC ribbon synapse, leading to a reduction of the SVs available for release (“standing RRP”) explaining the impaired synchronous activation of SGNs reflected by the reduced wave I in the auditory brainstem recording (ABR). Since RIM-BP2 is also knocked out in efferent olivocochlear neurons that provide inhibitory feedback onto the afferent terminals of type I SGNs (Ruel et al., 2001), we cannot rule out effects caused through alterations in efferent modulation. Nonetheless, the sound encoding phenotype upon RIM-BP2 disruption was rather mild. Future studies should test the presence and potential function of other RIM-BP isoforms (RIM-BP1 and RIM-BP3) that might partially compensate the function of RIM-BP2.

## Author contributions

SK, CW, and TM designed the study. SK performed immunohistochemistry and confocal/STED microscopy, patch-clamp capacitance measurements and Ca^2+^-imaging, and contributed to EM analysis, TB performed auditory nerve fiber recordings, SJ performed patch-clamp capacitance measurements and CW performed electron microscopy. TM and SK prepared the manuscript.

### Conflict of interest statement

The authors declare that the research was conducted in the absence of any commercial or financial relationships that could be construed as a potential conflict of interest.
